# Impact of carbon-based fibers morphologies on their carcinogenic potential

**DOI:** 10.1186/s12989-026-00663-y

**Published:** 2026-02-07

**Authors:** Anna Wagner, Florian Schulz, Asmus Meyer-Plath, Franziska Dahlmann, Susanne Rittinghausen, Dirk Schaudien

**Affiliations:** 1 Fraunhofer Institute for Toxicology and Experimental Medicine ITEM, Hannover, Germany; 2 Federal Institute for Occupational Safety and Health, Berlin, Germany

**Keywords:** Carbon, Carbon nanotubes, Fibers, Intraperitoneal, Mesothelioma

## Abstract

**Background:**

Carbon based fibers are considered to exhibit a carcinogenic potency when inhaled into the deep lung. Mesotheliomas develop after intraperitoneal application of multi-walled carbon nanotubes (MWCNTs) exceeding a diameter of about 37 nm, whereas carcinogenic potency decreases for diameters below this threshold. While large MWCNT diameters are associated with a rigid fiber geometry, this study examined the effects of MWCNTs with smaller diameters ranging from 10 to 30 nm. Also, a sample of single-walled carbon nanotubes (SWCNTs) exhibiting single fiber diameters significantly below 10 nm and showing a flexible geometry was included since individual SWCNT fibers can aggregate to form bundles that exhibit increased rigidity. Additionally, the carcinogenic effect of pitch-based carbon fiber fragments was investigated. Carbon fibers are industrially produced with diameters larger than 4 µm and are thus not per se respirable. However, pitch-based fibers tend to break along their longitudinal axis, resulting in respirable fragments, partially of critical WHO dimensions. Four CNT samples with a geometric mean diameter (GMD) of 30 nm, 20 nm, 10 nm, and smaller than 10 nm, as well as one fragmented carbon fiber sample (GMD 1.3 µm) were intraperitoneally injected into rats in two dosages (0.1 × 10^9^ and 1 × 10^9^ WHO fibers or WHO-analog nanofibers) and observed for up to 24 months. A long amosite asbestos (GMD 0.37 µm) with known fiber-specific carcinogenic effect served as a positive control (0.1 × 10^9^ WHO fibers).

**Results:**

A small number of mesotheliomas occurred in all fiber types, but not at all dosages. For the carbon fiber material, a possible weak carcinogenic potency is seen at the higher dosage. For the SWCNT fiber, low number of mesotheliomas likewise suggest a weak carcinogenic potency. In the case of the MWCNT fiber with a GMD of 30 nm, very low number of mesotheliomas indicate a possible very weak carcinogenic potency. No clear carcinogenic potency was observed for the MWCNTs with GMDs of 20 nm and 10 nm.

**Conclusions:**

Carbon fiber fragments and thin but bundled MWCNTs showed weak carcinogenic potency. Non-bundled MWCNTs with a diameter below 30 nm did not show clearcarcinogenic potency at a dose up to 1 × 10^9^ WHO-analog nanofibers.

**Supplementary Information:**

The online version contains supplementary material available at 10.1186/s12989-026-00663-y.

## Background

Carbon nanotubes (CNT) are produced industrially as single- or multiwalled fibers. Due to their high chemical stability, electrical and thermal conductivity as well as tensile strength at low density, they have found widespread application [1]. This increases the possibility of human and environmental exposure to such fibers during synthesis, processing, use and recycling [2, 3]. The respirability of fibers depends on their outer diameter. Therefore, CNTs with their outer diameter well below 1 µm are respirable unless excessive length well above 100 µm causes impaction in upper airways. CNTs are generally synthesized with a length exceeding 5 µm and, due to high chemical inertness, can show high biopersistence. Consequently, asbestos-like carcinogenicity in accordance with the fiber pathogenicity paradigm was anticipated [4].

Polyacrylonitrile (PAN)-based and mesophase pitch-based carbon fibers (CF) were developed in the early 1960s [5] and 1970s [6], respectively. CF are mostly used in composite materials as reinforcement or heat conductive additive [7]. CF are typically produced with diameters of 4.5–10 µm and are, therefore not initially respirable [8]. They are extreme rigid but also somewhat brittle. Mechanical or thermal stress and abrasion can lead to release of respirable fibers. In comparison to (PAN)-based CF, pitch-based CF show a higher tendency to split longitudinally, which can lead to thin respirable fibers fragments of critical dimension [9]. Until now, there are no long-term studies investigating the toxicologic potency of pitch-based CF.

An appropriate intraperitoneal test for carcinogenicity is accepted by the regulatory authorities in the EU as a non-physiological model to investigate whether a mineral wool fiber has the potential to induce pleural or peritoneal epithelial cell tumors (Regulation (EC) No 1272/2008 [10], JRC EUR 18748 EN [11]). The German Federal Institute for Occupational Safety and Health (BAuA) proposed that this test is also appropriate for other biopersistent fibers with fiber-related health concern [12]. Furthermore, an IP test may be preferable to a chronic inhalation study, as the latter could lead to false negative results although also potential false positive results can occur [13]. In the German regulation, the concept is already implemented for years and can be used for classification as carcinogen (TRGS 905 [14]). In this test, a suspension containing 10^9^ fibers of WHO dimensions is to be injected once into the peritoneum of each rat (for CF on a micrometer scale the term WHO fiber is used while for CNTs on a nanometer scale the term WHO-analog nanofiber is applied). Following this application, the animals are to be kept for 2 years and to be investigated histopathologically for the occurrence of mesotheliomas. All asbestos fiber types that caused mesotheliomas in humans as well as all asbestos and man-made mineral fiber types that caused mesotheliomas in experimental animals after inhalation exposure also caused mesotheliomas after intraperitoneal (*i.p.*) injection in rats [15–21]. Therefore, this test in rats is considered specific for the carcinogenicity of biopersistent fibers. In contrast to fibers, biopersistent granular dusts are not able to induce mesotheliomas in this test, even when applied at very high doses of 4 × 20 mg per rat [22]. A previous study showed that CNTs with a geometric mean diameter (GMD) exceeding 37 nm can induce mesotheliomas [23]. Studies that tested CNTs of smaller diameter in rats have not observed tumor genesis [24–26]. As only objects of fiber-like morphology are expected to show fiber-like pathogenicity, the rigidity of fibers came into focus to interpret diameter-dependent test results. Flexural rigidity is a physical quantity that scales with the fourth power of fiber diameter. It affects the morphology of an ensemble to be dominated by straight or non-rigid, floppy fibers appearance. Depending on growth mechanism, both rigid and non-rigid fibers can obtain wavy appearance and form tangled agglomerates already during synthesis, but non-rigid fibers can in addition spontaneously curl and entangle. Wavy fiber appearance or agglomeration state are therefore no reliable indicators of low rigidity. However, non-rigid fibers, which are flexible and can form tangled agglomerates, are considered to be less critical for the induction of mesotheliomas than rigid individual fibers [27]. Straight CNTs were found to cause stronger inflammatory reaction and by that pose a higher risk to cause mesotheliomas than tangled CNTs [28].

The 24-months study presented here was performed to determine the carcinogenic potential (especially incidence of mesotheliomas) of 4 different CNTs with a GMD between < 10 and 30 nm and a length greater than 5 µm as well as a sample of pitch-based carbon fiber fragments after intraperitoneal application in rats. All fiber samples were carefully characterized to investigate (I.) whether the previously established minimum MWCNT diameter threshold of 37 nm for asbestos-like carcinogenicity can be maintained or needs to be lowered, (II.) the carcinogenic potential of SWCNT and (III.) the carcinogenic potential of microscale carbon fiber fragments.

In addition to the long-term study over 2 years, inflammatory effects of the different fibers in the peritoneum were investigated 3 months after *i.p.* application to explore whether such a subchronic test can be predictive for future mesothelioma development.

## Methods

### Aim of the study

This study was performed to address open questions regarding the principles of the carcinogenic effect of micro- and nanoscale carbonaceous fibers with WHO dimensions. A specific focus was put on the influence of fiber rigidity and GMD on carcinogenicity.

1. Can very thin, highly flexible SWCNT with diameters of below 10 nm, which can be present both in single and agglomerate form, including fiber-shaped bundles, can have an asbestos-like effect?
2. In previously published studies on *i.p.* tests with carbon nanotubes thinner than 20 nm, only comparatively short fibers with lengths < 5 µm were used and no asbestos-like effect was found. It was therefore investigated whether such fibers can have asbestos-like effects if they are administered in WHO fiber dimensions.
3. Do asbestos-like effects only occur above the previously determined critical diameter of 37 nm, or could it be necessary to lower this critical diameter of carbon nanotubes to 30 nm or below?
4. Do carbon fiber fragments with WHO fiber dimensions, which may also occur at workplaces due to mechanical stressing, induce an asbestos-like effect?

In preparation of the present study, the BAuA (Berlin and Dortmund, Germany) performed an initial market research, including batch characterization, if materials meeting those criteria are commercially available. For the aim of investigating the influence of fiber rigidity, it was considered important for the diameter distribution of the material candidates to be as narrow as possible. This initial market study did not reveal appropriate CNT products in the diameter range between 10 and 30 nm. Therefore, some of the test materials were specifically synthesized for this study. One synthesis was carried out by the Leibniz Institute for Solid State and Materials Research (IFW, Dresden, Germany). Another, NC-7000 long, was a research-grade material provided by Nanocyl SA, Sambeville, Belgium. This resulted in the material selection shown in Table 1. The pitch-based carbon fiber sample of type Dialead K13D2U was micronized at BAuA by ball-milling as described below.

**Table 1:** Overview of material selection for this study

Material class	1	2	3	4	5
Manufacturer	OCSiAl	Nanocyl	IFW	USRN	Mitsubishi
Product	Tuball	NC-7000 long*	CNT1-1*	USRN 20–30	Dialead K13D2U
Specification	Single- walled carbon nanotubes (SWCNT) with diameters below 10 nm	Diameter distribution as close to 10 nm as possible	Diameter distribution as close to 20 nm as possible	Diameter distribution as close to 30 nm as possible	Pitch-based carbon fiber fragments with WHO fiber dimensions
Characteristics	Non-rigid SWCNT, some present as compact, bundled fiber agglomerates with a high aspect ratio	Most likely non-rigid multi-walled carbon nanotubes (MWCNT) with GMD F_WHO_ ~ 10 nm	Possibly rigid MWCNT with GMD F_WHO_ ~ 20 nm	Possibly rigid MWCNT with GMD F_WHO_ ~ 30 nm	Rigid carbon-based fibers

Materials marked by asterisk* were research-grade materials. *GMD* geometric mean diameter. *F*_*WHO*_ WHO fibers or WHO-analog nanofibers

OCSiAl Europe S.à.r.l., Leudelange, Luxembourg; Nanocyl SA, Sambreville, Belgium; IFW, Leibniz Institute for Solid State and Materials Research, Dresden, Germany; USRN: US Research Nanomaterials, Inc., Houston, Texas, USA; Mitsubishi Chemical Corporation (Tokyo, Japan)

The class 5 fiber material were fragments of a mesophase pitch-based carbon fiber of type Dialead K13D2U (Mitsubishi). The GMD for the WHO fiber fraction of 1.2 µm should render them rigid. Carbon fibers are typically manufactured with diameters of 7–10 µm and are therefore not initially respirable. Mechanical failure can disintegrate the fibers into respirable fragments [29]. Here, disintegration was achieved by ball-mill grinding for 2 × 45 s at 5000 rpm using a Precellys 24 tissue homogenizer (Bertin Instruments, Montigny-le-Bretonneux, France) equipped with 24 lysing vials of 2 mL volume of type CK68-R, each containing two ceramic balls of 6.8 mm diameter and 110 mg of carbon fiber. Since Long Amosite was only available as raw material, further grinding and characterization was necessary to obtain material with a comparable GML.

### Characterization of test items

**Fig. 1:**
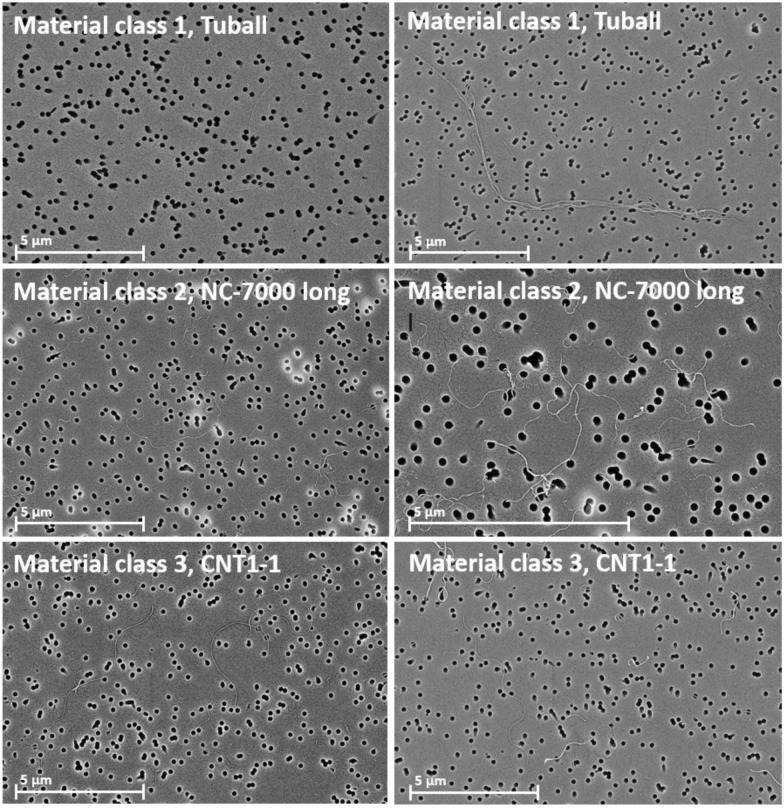
Exemplary images of fibers of material classes 1, 2 and 3

**Fig. 2:**
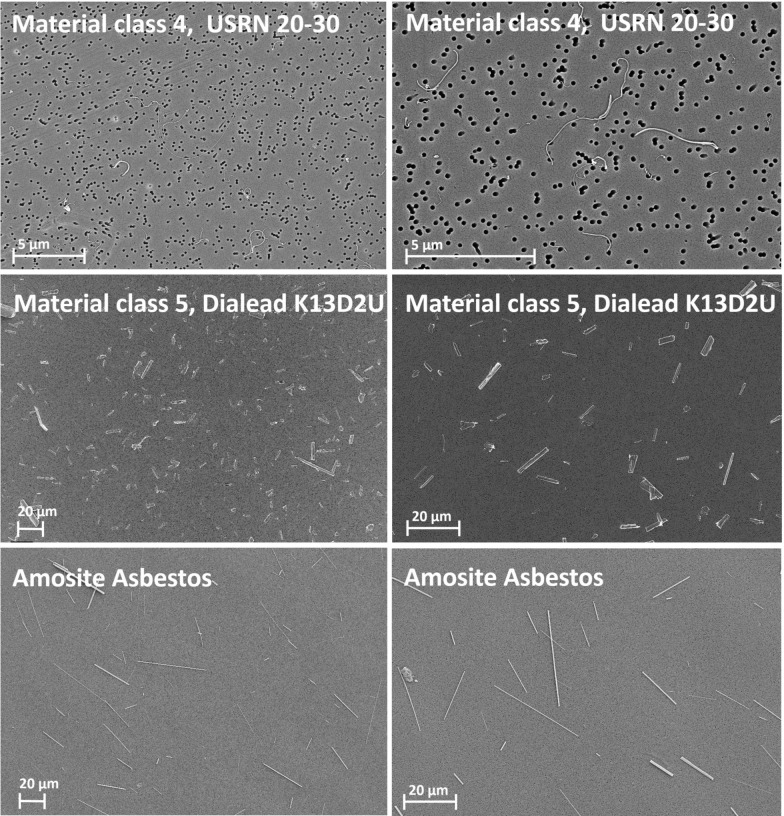
Exemplary images of fibers of material classes 4, 5 and Amosite Asbestos

For the characterization, the test materials were dispersed in Porter’s dispersion medium [30], 500 mL PBS (Ca and Mg free)–Sigma-Aldrich #D8537, 495 mg alpha-D-Glucose—Sigma Aldrich #158968, 300mg Bovine Serum Albumin—Sigma-Aldrich #A9647, 5mg DPPC—Sigma Aldrich #P0763). This medium is very similar to the physiological surfactant-containing environment in the lungs, facilitates the suspension of the fibers and is not toxic to the animals. To achieve the best possible separation of the test materials in the medium, they needed to be ultrasonicated with an ultrasonic tip (Bandelin electronic Sonopuls HD 2070 ultrasonic homogenizer with sonotrode VS 70 T, Berlin, Germany). A treatment of 10 min duration at an intensity of 100% was applied for OCSiAl Tuball. It was effective in dispersing larger agglomerates, but some bundled individual fibers were still present, see example images in Fig. 1. For Nanocyl NC-7000 a duration of 1 min at an intensity of 100% was sufficient to separate agglomerated fibers effectively. For CNT1-1, a duration of a few seconds at an intensity of 100% and then 1 min at an intensity of 30% was applied and a duration of a few seconds at an intensity of 100% and then 2 min at an intensity of 30% was used for USRN 20–30. For dispersion of the carbon fiber fragments, the most suitable method was stirring for 18 h on a magnetic stirrer, followed by a 15-min treatment in an ultrasonic bath (Bandelin electronic Sonorex RK 510 H, Berlin, Germany) and a 2-min treatment with the ultrasonic tip at 100% (see example images in Fig. 2). Long Amosite was ground with a Moulinex mill (Groupe SEB WMF Consumer GmbH, Geislingen/Steige, Germany) for 30 s. Dispersing was carried out by ultrasonic tip for 2 × 5 min at 100%.

The suspended fiber samples were filtered onto nuclepore filters (25 mm diameter, pore size 0.2 μm), sputter-coated with a layer of gold of about 20 nm thickness (Safematic CCU-010, Safematic GmbH, Switzerland) and analyzed by scanning electron microscopy (SEM, Zeiss Supra 55, Carl Zeiss AG, Oberkochen, Germany). All measurements were carried out based on the WHO/EURO method (WHO 1985). The fiber length and diameter of all evaluated objects were determined at a magnification of at least 2000x, the diameter at a magnification of up to 300,000x. Objects with a length-to-diameter aspect ratio of at least 3:1 were counted as fibers. All other objects were considered particles or agglomerates. This includes fiber fragments with an aspect ratio below 3:1. All objects within a field of view were counted. Fibers that protruded beyond the image section to be analyzed were counted as half fiber if there was only one fiber end within the field of view and were not counted if both ends were outside the field of view. In the initial characterization at least 100 WHO fibers or WHO-analog nanofibers and 50 fibers in the category shorter than 5 µm were counted. At least 200 WHO fibers or WHO-analog nanofibers and 100 fibers shorter than 5 µm were characterized in the suspensions used for intraperitoneal (*i.p.*) administration in the carcinogenicity study. Fiber counts were normalized to the inspected filter area and multiplied with the ratio of total filter area to filtered suspension volume to estimate fiber concentrations in suspension.

Biopersistent fibrous objects that are longer than 5 µm and have a diameter of < 3 µm have been reported to exhibit asbestos-like effects. If similar effects also occur for fibers of shorter length, for example in the range of 3.5–5 µm, is not fully understood. Therefore, assessing toxicological results of fibers requires an extensive size characterization to study dose–effect relationships. As part of the comprehensive characterization of our material samples, all fibrous objects, i.e. fibers and fiber agglomerates with aspect ratios greater than 3 were classified into the following four length classes: shorter than 3.5 µm, between 3.5 and 5 µm, between 5 and 20 µm and larger than 20 µm. The latter two are comprised in the category WHO-analog nanofibers or WHO fibers, if the diameter is below 3 µm.

Due to the large number of animals, it was necessary to prepare several separate suspensions for the high-dose groups, as the suspension volume was limited to 30 mL. All these suspensions were characterized separately. In the low-dose groups and for OCSiAl Tuball, however, dilution from a stock solution was carried out. Therefore, there is only one individual value for these samples.

### Dosing scheme

Two different fiber concentrations were used in this study, 0.1 × 10^9^ (low dose) and 1 × 10^9^ (high dose) WHO fibers. In a recent study [31], higher CNT fiber concentrations (1 × 10^9^ and 5 × 10^9^ WHO fibers) had been used which induced mesotheliomas in the peritoneal cavity on an early timepoint with high mortality rates. Therefore, lower fiber concentrations were used in this study. Amosite asbestos was chosen as positive control and injected in a concentration of 0.1 × 10^9^ WHO fibers. With this concentration, amosite asbestos had provoked about 50% mesothelioma-bearing rats in 2-year studies [16, 31].

Dosing was performed according to the schedule in Table 2. Fiber concentrations in mg per 2 mL dispersion medium were calculated based on respective F_WHO_ concentrations per mg material as reported in Supplementary Table S1 for the final preliminary test batch. For the 2-year study, 50 animals per group were treated with a single intraperitoneal (*i.p.*) injection of 2 mL of the positive control amosite asbestos or the CNT-dispersion, respectively. Only the carbon fiber fragments (ground Dialead K13D2U) had to be injected twice on two consecutive days, because a comparably large amount of test material had to be dispersed. The dispersion medium served as negative control. For the 3-month study, additional 5 animals of all low dose groups as well as positive and negative control groups were treated with the test substances, amosite asbestos and dispersion medium, respectively.

**Table 2:** Overview of the intended dose groups for the carcinogenicity study

Group	Treatment	Dose [× 10^9^ F_WHO_]	Dose [mg/2 mL DM]	Number of rats per group
1	Dispersion medium (negative control)	–	–	55
2	Long amosite (positive control)	0.1	0.65	55
3	Fragmented carbon fiber (Dialead K13D2U)	0.1	2 × 1.45 (2.90)^#^	55
4		1	2 × 14.5 (29.0)^#^	50
5	CNT1-1* (MWCNT)	0.1	0.032	55
6		1	0.321	50
7	USRN 20–30 (MWCNT)	0.1	0.066	55
8		1	0.66	50
9	OCSiAl Tuball (SWCNT)	0.1	0.0009	55
10		1	0.009	50
11	Nanocyl NC7000 long* (MWCNT)	0.1	0.007	55
12		1	0.07	50
Total number of animals				635

^*^Research-grade material

^#^Fibers with a GML > 5 µm were used to calculate the sample weight. This also included fibers with a diameter ≥ 3 µm to a minor extent. The actual nominal dose for the carbon fiber was therefore 0.93 or 0.093 × 10^9^ WHO fibers. F_WHO_ refers to WHO-analog nanofibers in the case of CNTs and to WHO fibers in the case of the carbon fiber and the positive control

### Animal experiment

The study was approved according to the German Animal Welfare Act by the local authority at the LAVES Niedersachsen, Hannover, Germany, No. 33.19-42502-04-17/2683.

For the 24 months study, 600 male 10-weeks old Wistar Han rats Crl:WI(Han) (Charles River Deutschland, Sulzfeld, Germany), 50 for each group, were used. In addition, 35 rats were utilized for the 3 months study, 5 for each low dose group and both control groups.

Animals were housed in groups of three rats in polycarbonate cages (Macrolon^®^ type IV) on absorbent softwood bedding (Lignocell BK 8–15, JRS GmbH & Co KG, Rosenberg, Germany). The rats got tap water (Stadtwerke Hannover, Germany) and pelleted food (R/M-H V1534, Ssniff, Soest, Germany) ad libitum fresh weekly or more often, if necessary. Standard animal housing conditions consisted of a temperature of 20 ± 2 °C and a 12-h light/dark cycle. Unequivocal animal identification was ensured by tattooing the ears. Animal checking for clinical symptoms, morbidity, or mortality was performed at least once daily. Body weight was measured at weekly intervals. Rats were kept for 3 months or 24 months after *i.p.* treatment, respectively, and sacrificed at the end of the study. However, morbid rats were previously euthanized to avoid unnecessary pain and suffering. Histopathological examination of the deceased animals was done analogous to animals regularly killed at the end of the experiment.

### Histology

Full gross necropsy was performed on all animals, including those which died during the experiment or were killed in a moribund condition. Organs and pathological lesions were fixed in 10% buffered formalin for 24 h. If trimming was possible only after a weekend, the tissue material was transferred and fixed in 70% ethanol for up to 48 h. In all animals, tissue samples of the diaphragm, omentum, intestinal mesenteries plus gut segment, liver (with falciform ligament), spleen, pancreas and, except for the animals of the interim sacrifice, scrotum with at least one testis were processed for histopathology. In addition, all macroscopic lesions of the abdominopelvic cavity and genital tract were prepared. Tissue masses outside the abdominal cavity were only examined if they were macroscopically suspicious of tumors. After embedding of tissues in paraffin, 3-µm thick sections were prepared and routinely stained with hematoxylin–eosin. Slides of all tissues were examined by a certified veterinary pathologist using a bright-field microscope and the INHAND (International Harmonization of Nomenclature and Diagnostic Criteria for Lesions in Rats and Mice) nomenclature for classification of tumors and pre-neoplastic lesions of the mesothelium [23] was applied. A peer-review of tumors and borderline lesions was done by a second certified veterinary pathologist. A 5-part severity score was used to assess the severity of non-neoplastic lesions (1 = very slight; 2 = slight; 3 = moderate; 4 = severe; 5 = very severe).

### Immunohistochemical analysis

For further classification of tumors of the peritoneum or tunica vaginalis, immunohistochemical staining of representative slides was performed. The primary antibodies used to characterize mesotheliomas and to compare them with mesotheliomas in humans comprised monoclonal mouse anti-vimentin (clone V9, isotype IgG1 kappa, Dako Denmark A/S, 2600 Glostrup, Denmark, #M 0725), monoclonal mouse-anti-pan-cytokeratin (clone PCK-26, Sigma-Aldrich Co. LLC, Saint Louis, Missouri 63103, USA, #C-1801), and mouse-anti-podoplanin (clone LF3, Novus Biologicals, Cambridge, UK, #NB110-96423). Furthermore, anti-smooth muscle actin, anti-S-100, anti-CD68 (CD68/ED1 Serotec MCA 341R) and in selected cases anti-CD3, anti-CD79alpha and anti-Ki-67 were used to exclude or confirm other tumors or hyperplastic lesions. For immunohistochemical detection of the markers, 3-µm-thick paraffin sections were cut from chosen blocks containing the tumor and mounted on glass slides. Afterwards, dewaxing of paraffin sections was performed followed by specific antigen retrieval procedure for each of the primary antibodies. Heat-induced antigen retrieval was performed in a citrate-buffered solution. After rinsing with Tris-buffered saline (pH 7.6) plus 0.01% Tween^®^20 (Merck KGaA, Darmstadt, Germany, 8.22184), slides were incubated in normal goat serum (Vector Laboratories Inc., Burlingame, CA, USA, S-1000) for 20 min at 21 °C and then with the primary antibody for 1 h at 21 °C. This was followed by incubation for 30 min at 21 °C with the secondary antibody biotin-SP-conjugated AffiniPure goat anti-mouse IgG (Jackson Immunoresearch, West Grove, PA, USA, 111-065-100). For immunostaining, a routine method was performed using alkaline phosphatase-streptavidin–biotin (Vector Laboratories Inc., Burlingame, CA, USA, #S-5100) and Fast Red as chromogen (Fast Red Substrate Package, BioGenex, Freemont, CA, USA, #HK182-5 K). Finally, slides were counterstained with Mayer’s hematoxylin (Linaris Biologische Produkte GmbH, Wertheim-Bettingen, Germany, EGH3411) and mounted with the aqueous embedding medium Aquatex^®^ (Merck KGaA, Darmstadt, Germany, #1.08562).

### Measurement of peritoneal thickness

The tissue samples used for histopathologic analysis were digitized via the NanoZoomer S210 (Hamamatsu Photonics, Herrsching am Ammersee, Germany). An algorithm was developed to identify the diaphragmatic serosal tissue, to measure the serosal tissue area as well as the length of the serosal tissue area utilizing the Visiopharm image analysis software (Visiopharm A/S Hørsholm, Denmark). The thickness was calculated by dividing the serosal tissue area with the serosal tissue length.

### Statistics

The number of rats with and without treatment-related findings in different dose groups was compared with the medium control group by the chi-squared & Fisher’s Exact test, choosing a level of significance of p ≤ 0.05 using the Provantis Software (Version 10.5.0.3, Instem^®^, Stone, Staffordshire, United Kingdom). The statistical analysis of the diaphragmatic serosal thickness measurement was done with the Statistica software (Version 13, StatSoft GmbH, Hamburg, Germany) using analysis of variance (ANOVA) for an initial analysis.

## Results

### SEM characterization of the test materials

#### Material class 1 (SWCNT OCSiAl Tuball)

This fiber material was selected as non-rigid single-walled carbon nanotube (SWCNT). However, the sample (OCSiAl Tuball) with a nominal fibril diameter of 1.7 nm contained not only individual SWCNT fibrils but also high aspect ratio fiber bundles with a rope-like structure and compact appearance. Such bundles are known to form already during the CVD synthesis, and, due to high van-der-Waals interaction forces, it requires special solvents and high energy input to separate the majority of SWCNT fibrils. No such measures were applied here.

For the high-dose group, a GMD of around 7 nm and a GML of 6.76 µm were obtained for the fraction of WHO-analog nanofibers (see Table 3, supplementary Table S2.1). In the low-dose group, values for the GMD of around 8 nm and for the GML of 6.89 µm were determined. However, the GMD should only be considered an approximate value, as it was at the edge of the SEM resolution limit. Thus, also the number of single fibers may be underestimated, and the dose of WHO-fibers may be more referred to fiber bundles. However, if fiber bundles with rope-like structures and enhanced rigidity pose more likely health concern, these objects may be a more relevant dose matrix to be tested with a dose of 1 × 10^9^ WHO fibres. The relative proportion of WHO-analog nanofibers was about 8% in the high dose group and about 13% in the low dose group. The largest proportion of the test material were fibers with a GML of less than 3.5 µm (> 75%). Fibers with a GML between 3.5 and 5 µm were also present to a certain extent. The proportion of particles was negligible. Percentile values concerning length and diameter regarding all CNT materials are provided in supplementary Table S3 for the categories all fibers, WHO-analog nanofibers and particles. For OCSiAl Tuball only one particle/agglomerate was found in the SEM fields of view used for evaluation. This object was clearly in the respirable range. The actual number administered to the animals in the low dose group was about 0.08 × 10^9^ WHO-analog nanofibers, slightly below the target number of 0.1 × 10^9^ WHO-analog nanofibers (see Table 3, supplementary Table S2.1). In the high dose group, the target of 1 × 10^9^ WHO-analog nanofibers was almost exactly achieved with the number of 1.06 × 10^9^ WHO-analog nanofibers.

**Table 3:** Actual injected carbon nanotubes and amosite asbestos doses

		OCSiAl Tuball	OCSiAl Tuball	Nanocyl NC-7000 long*	Nanocyl NC-7000 long*	IFW CNT1-1*	IFW CNT1-1*	USRN 20-30	USRN 20–30	Long Amosite
		Low	High	Low	High	Low	High	Low	High	
Concentration	Target concentration/animal	0.1 × 10^9^	1 × 10^9^	0.1 × 10^9^	1 × 10^9^	0.1 × 10^9^	1 × 10^9^	0.1 × 0^9^	1 × 10^9^	0.1 × 10^9^
	Number F_WHO_/mg material	83.8 × 10^9^	113.8 × 10^9^	15.2 × 10^9^	10.8 × 10^9^–15.9 × 10^9^	2.4 × 10^9^	2.3 × 10^9^–2.7 × 10^9^	1.5 × 10^9^	1.2 × 10^9^–1.4 × 10^9^	0.14 × 10^9^
	**Actual concentration/animal**	**0.08 × 10**^**9**^	**1.06 × 10**^**9**^	**0.11 × 10**^**9**^	**0.78 × 10**^**9**^–**1.15 × 10**^**9**^	**0.08 × 10**^**9**^	**0.74 × 10**^**9**^** – 0.85 × 10**^**9**^	**0.10 × 10**^**9**^	**0.78 × 10**^**9**^**–0.94 × 10**^**9**^	**0.09 × 10**^**9**^
Relative percentage	F_WHO_	13.51%	8.27%	5.31%	2.53–3.56%	4.00%	2.86–4.41%	3.10%	1.46–2.30%	57.80%
	3.5 < = L < = 5 µm	10.86%	2.12%	10.25%	3.75–9.15%	3.75%	3.81–6.09%	5.21%	1.40–5.36%	14.56%
	< 3.5 µm	75.52%	89.57%	83.07%	86.88–92.19%	87.18%	85.32–90.88%	90.50%	93.13–97.09%	26.20%
	L/D < 3 (Agglom./Part.)	0.11%	0.03%	1.36%	0.73–1.06%	5.07%	2.43–4.18%	1.18%	0.00–0.17%	1.44%
PSD F_WHO_	**GML (µm)**	**6.89**	**6.76**	**6.53**	**6.22–6.50**	**7.42**	**7.17–7.71**	**6.72**	**6.66–6.84**	**12.93**
	SD	1.27	1.32	1.27	1.18–1.28	1.35	1.37–1.42	1.32	1.28–1.35	1.96
	**GMD (µm)**	**0.008**	**0.007**	**0.012**	**0.010–0.011**	**0.020**	**0.018–0.020**	**0.027**	**0.029–0.030**	**0.371**
	SD	1.526	1.505	1.431	1.331–1.515	1.483	1.417–1.482	1.53	1.090–1.127	1.734
PSD 3.5 < L < 5 µm	GML (µm)	4.29	4.17	4.08	3.97–4.12	4.02	3.99–4.49	4.00	3.82–4.21	4.11
	SD	1.1	1.12	1.11	1.09–1.14	1.05	1.06–1.14	1.13	1.05–1.11	1.12
	GMD (µm)	0.007	0.009	0.013	0.006–0.01	0.016	0.017–0.022	0.039	0.028–0.034	0.311
	SD	1.444	1.433	1.539	1.274–1.342	1.582	1.335–1.724	1.248	1.000–1.128	1.885
PSD L/D > 3 (All fibers)	GML (µm)	1.65	1.27	1.35	1.09–1.37	1.25	1.09–1.26	1.3	0.93–1.15	6.77
	SD	2.64	2.43	2.22	1.99–2.17	2.05	2.01–2.17	2.05	1.90–2.07	2.61
	GMD (µm)	0.007	0.007	0.011	0.007–0.010	0.018	0.015–0.018	0.025	0.023–0.026	0.322
	SD	1.528	1.528	1.494	1.374–1.526	1.464	1.435–1.469	1.485	1.268–1.395	1.809

F_WHO_ refers to WHO-analog nanofibers in the case of CNTs and to WHO fibers in the case of carbon fibers and the positive control; important parameters are given in bold

Agglom: agglomerates; Part.: particles; L: length, D: diameter; PSD: particle size distribution; GML: geometric length; GMD: geometric diameter; SD: standard deviation; * research-grade materials

#### Material class 2 (MWCNT Nanocyl NC-7000 long)

This fiber material was multi-walled carbon nanotubes (MWCNT) (Nanocyl NC-7000 long) and considered to be most likely non-rigid. Nanocyl NC-7000 long was a research-grade material. The WHO-analog nanofibers in the high-dose group showed a GMD of 10–11 nm and a GML of 6.2–6.5 µm of the fibers were measured (Table 3, supplementary Table S2.2). In the low-dose group, values were determined for the GMD of 12 nm and for the GML of 6.5 µm. The relative proportion of WHO-analog nanofibers was about 5% in the low dose group and about 3% in the high dose group. The largest proportion of the test material were fibers with a GML of less than 3.5 µm (> 83%). Fibers with a GML between 3.5 and 5 µm were also present to a certain extent (about 4–10%). The proportion of particles was about 1%. No particles/agglomerates outside the respirable range were found (Supplementary Table S3). The number administered in the low dose group was 0.11 × 10^9^ WHO-analog nanofibers, which was almost identical to the target number of 0.1 × 10^9^ WHO-analog nanofibers, see Table 3 and supplementary Table S2.2. In the high dose group (target concentration 1 × 10^9^ WHO-analog nanofibers), the values in the dispersions were between 0.78 and 1.15 × 10^9^ WHO-analog nanofibers.

#### Material class 3 (MWCNT CNT1-1)

This fiber material (CNT1-1) was a MWCNT specifically synthesized for this test by the Leibniz Institute for Solid State and Materials Research (IFW, Dresden, Germany) and was considered to be possibly rigid. In the dispersions for the high-dose group, GMD values of 18 to 20 nm and a GML between 7.2 and 7.7 µm were determined for the fraction of WHO-analog nanofibers (Table 3, supplementary Table S2.3). In the low-dose group, the GMD was 20 nm and the GML 7.4 µm. The relative proportion of WHO-analog nanofibers was about 3 to 4% in the low and high dose group. The largest proportion of the test material were fibers with a GML of less than 3.5 µm (> 87%). Fibers with a GML between 3.5 and 5 µm were also present to a certain extent (about 4–6%). The proportion of particles was about 3–5%. No particles/agglomerates outside the respirable range were found (Supplementary Table S3). The number administered in the low dose group was approximately 0.08 × 10^9^ WHO-analog nanofibers, which was slightly below the target number of 0.1 × 10^9^ WHO-analog nanofibers (Table 3, supplementary Table S2.3). In the high dose group, the values of 0.74 to 0.85 × 10^9^ WHO-analog nanofibers were also slightly below the target concentration of 1 × 10^9^ WHO-analog nanofibers.

#### Material class 4 (MWCNT USRN 20–30)

This fiber material was MWCNT (USRN 20–30) considered to be possibly rigid. In the dispersions for the high-dose group, the GMD of the WHO-analog nanofibers was between 27 and 30 nm. The GML ranged from 6.66 to 6.84 µm (Table 3, supplementary Table S2.4). In the low-dose group, values were determined for the GMD of 27 nm and for the GML of 6.72 µm. The relative proportion of WHO-analog nanofibers was about 3% in the low dose group and 1.5–2.3% in the high dose group. The largest proportion of the test material were fibers with a GML of less than 3.5 µm (> 90%). Fibers with a GML between 3.5 and 5 µm were also present to a certain extent (1.4–5.4%). The proportion of particles was 1% or below. No particles/agglomerates outside the respirable range were found (Supplementary Table S3). The number administered in the low dose group was exactly the target value of 0.1 × 10^9^ WHO-analog nanofibers (Table 3, supplementary Table S2.4). In the high dose group, the values were 0.78–0.94 × 10^9^ WHO-analog nanofibers, slightly below the target concentration of 1 × 10^9^ WHO-analog nanofibers.

#### Material class 5 (Carbon fiber fragments Dialead K13D2U)

In the dispersions for the high-dose group, GMD values in the range of 1.22–1.39 µm and GML of 6.78–7.51 µm were obtained (Table 4, supplementary Table S2.5). In the low-dose group, values for the GMD of 1.25 and 1.27 µm and for the GML of 7.13 and 7.37 µm were determined. The relative proportion of WHO fibers was in the range of 30% in the low and high dose group. Fibers with a GML between 3.5 and 5 µm ranged from 15 to 20%. The proportion of fibers with a GML of less than 3.5 µm was about 10–15% in most of the suspensions. The proportion of particles was about 25–40%. Percentile values concerning length and diameter regarding all CNT materials are provided in supplementary Table S4 for the categories all fibers, WHO fibers, particles as well as fibers longer than 5 µm irrespective of the diameter (including also fibers thicker than 3µm). The 95th percentile values were below 3 µm in the category all fibers. In the category particles also some objects with a 95th diameter percentile above 3 µm were found. The 90th percentile was below 3 µm in most suspensions. This was the best achievable material as further milling would have caused loss of WHO fibers. It reflects also the dust at the workplace, and such particles would also be respirable for humans. The actual number of WHO fibers administered in the low dose group was 0.08 × 10^9^ WHO fibers, which is slightly below the target value (Table 4, supplementary Table S2.5). In the high dose group, the values were in the range between 0.74 and 0.97 × 10^9^ WHO fibers.

**Table 4:** Actual injected carbon fiber doses

		Mitsubishi Dialead K13D2U fragments	Mitsubishi Dialead K13D2U fragments
		Low	High
Concentration	Target concentration/animal	0.1 × 10^9^	1 × 10^9^
	Number F_WHO_/mg material	0.029 × 10^9^ and 0.027 × 10^9^	0.023 × 10^9 −^ 0.036 × 10^9^
	**Actual concentration/animal**	**0.08 × 10**^**9**^	**0.74–0.97 × 10**^**9**^
Relative percentage	Fibers L > 5µm	36.5%/ 43.8%	30.6–42.3%
	F_WHO_	30.1%/ 36.6%	27.0–38.1%
	Fibers L > 5µm, D ≥ 3 µm	6.4%/7.1%	3.6–5.4%
	3.5 < = L < = 5 µm	15.3%/19.8%	14.5–19.5%
	< 3.5 µm	14.2%/9.9%	9.0–19.0%
	L/D < 3 (Agglom./Part.)	34.0%/26.6%	25.4–40.4%
PSD F_WHO_	**GML (µm)**	**7.13/7.37**	**6.78**–**7.51**
	SD	1.32/1.31	1.28–1.35
	**GMD (µm)**	**1.245/1.269**	**1.217**–**1.3850**
	SD	1.582/1.465	1.4560–1.5890
PSD 3.5 < L < 5 µm	GML (µm)	4.16/4.25	4.18–4.32
	SD	1.11/1.12	1.10–1.12
	GMD (µm)	0.827/0.818	0.788–0.916
	SD	1.384/1.64	1.3710–1.590
PSD L/D > 3 (All fibers)	GML (µm)	5.20/5.71	4.79–5.70
	SD	1.68/1.60	1.55–1.64
	GMD (µm)	1.007/1.064	0.904–1.118
	SD	1.769/1.750	1.593–1.807

*The carbon fiber was administered twice per animal to achieve the target concentration

F_WHO_ refers to WHO-analog nanofibers in the case of CNTs and to WHO fibers in the case of carbon fibers and the positive control; important parameters are given in bold

*Agglom* agglomerates, *Part.* particles,* L* length, *D* diameter, *PSD* particle size distribution, *GML*: geometric length, *GMD*: geometric diameter, *SD*: standard deviation

#### Positive control (Long amosite)

The positive control was applied only as low dose with a target concentration of 0.1 × 10^9^ WHO fibers. The GMD was 0.37 µm and the GML 12.93 µm (Table 3, supplementary Table S2.6). The relative proportion of WHO fibers was 58%. Fibers with a GML between 3.5 and 5 µm were present with 15%. The proportion of fibers with a GML of less than 3.5 µm was about 26% in most of the suspensions. The proportion of particles was about 1%. No particles/agglomerates outside the respirable range were found (Supplementary Table S3). The number of 0.09 × 10^9^ WHO fibers applied was only slightly below the anticipated value (Table 3, supplementary Table S2.6).

#### Body weight development

The development of body weight is shown in Supplementary Fig. S1. Only the body weights of the positive control showed a statistically significant reduction in body weight over the later course compared to the vehicle control group (Anova & Dunnett’s Test, 2 Sided). All other groups did not differ significantly from the vehicle control group.

#### Mortality

**Fig. 3:**
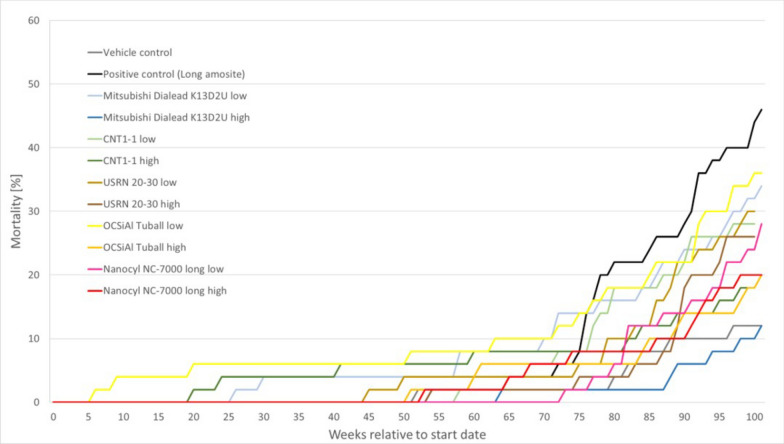
Cumulative mortality over the 24-months investigation period

The development of cumulative mortality depending on the course of the study is shown in Fig. 3.

Only the animals that were removed from the experiment prematurely due to a moribund state are listed here. In several groups, animals had to be removed from the experiment prematurely (test duration < 500 days). The main reasons for this were various neoplasms (e.g. malignant lymphomas, malignant schwannomas, fibrosarcomas) or inflammatory changes in various organ systems (e.g. purulent-necrotizing prostatitis, chronic active enteritis), which manifested themselves clinically in weight loss, poor general condition, or externally visible and palpable masses. Older animals that had to be removed from the experiment predominantly showed neoplasms, which in most cases were not related to the fiber treatment. The different mortality rates in the non-asbestos groups are therefore not related to the fiber treatment.

Most animals with mesotheliomas (except of the amosite asbestos group) were necropsied at the end of the regular study. In only 2 cases a mesothelioma was the cause for early removal of the animals from the experiment (1 animal in group 8 after 637 days, 1 animal in group 10 after 716 days). Furthermore, 2 animals were removed from the experiment early due to other neoplasms (fibroadenoma of the mammary gland, thyroid carcinoma), in which a mesothelioma was also diagnosed.

#### Macroscopic findings

After 3 months, in 2/5 animals of the amosite asbestos group thickening of the ligamentum falciforme was observed macroscopically. Most of the animals of the CNT and CF treatment groups (except group 9) showed small black aggregates on the serosal surface of the abdominal cavity.

At necropsy of the 24-months study, in many animals of the amosite asbestos group and in few rats of the CNT and CF treatment groups ascites were discovered consisting of blood-containing fluid. Other frequent lesions in the amosite asbestos group comprised adhesions and multifocal to coalescing small nodules on the surface of the abdominal organs and tunica vaginalis, as well as thickening of the capsule of the spleen. These findings were also present in some animals of the CNT and CF treatment groups. However, the majority of CNT and CF treated animals (except animals of group 9) revealed under 1mm small aggregates of black foreign material covered by a thin layer of serosa, throughout the abdominal cavity and tunica vaginalis. This finding was more exaggerated than in the animals of the subchronic study.

#### Histopathological findings

##### Mesotheliomas

In the animals of the 3-months study no neoplastic lesions were present.

50 animals of all groups were examined for mesotheliomas in the abdominal cavity 2 years after the CNT injection. Incidences of mesotheliomas as well as their localization and subtypes are described in Tables 5 and 6, respectively.

**Table 5:** Occurrence of mesotheliomas in the carcinogenicity study

	Medium control	Amosite asbestos	Dialead K13D2U Carbon fiber fragments		CNT1-1 MWCNT		USRN 20–30 MWCNT		OCSiAl Tuball SWCNT		Nanocyl NC7000 long MWCNT	
			Low	High	Low	High	Low	High	Low	High	Low	High
**Group**	**1**	**2**	**3**	**4**	**5**	**6**	**7**	**8**	**9**	**10**	**11**	**12**
Number of animals examined	(50)	(50)	(50)	(50)	(50)	(50)	(50)	(50)	(50)	(50)	(50)	(50)
Mesotheliomas peritoneum and possibly tunica vaginalis	0	16	0	2	0	0	1	1	1	1	1	0
Mesotheliomas only tunica vaginalis	0	1	0	2	1	0	1	0	0	1	0	0
Mesotheliomas, early-stage Tunica vaginalis	0	0	0	1	1	0	0	0	0	2	0	0
**Mesotheliomas, total**	**0**	17***	**0**	**5**	**2**	**0**	**2**	**1**	**1**	**4**	**1**	**0**

Chi-square four-way test: * = p < 0.05; ** = p < 0.01; *** = p < 0.001

**Table 6:** Subtyping of mesotheliomas in the carcinogenicity study

	Medium control	Amosite asbestos	Dialead K13D2U Carbon fiber fragments		CNT1-1 MWCNT		USRN 20–30 MWCNT		OCSiAl Tuball SWCNT		Nanocyl NC7000 long MWCNT	
			Low	High	Low	High	Low	High	Low	High	Low	High
**Group**	**1**	**2**	**3**	**4**	**5**	**6**	**7**	**8**	**9**	**10**	**11**	**12**
Number of animals examined	(50)	(50)	(50)	(50)	(50)	(50)	(50)	(50)	(50)	(50)	(50)	(50)
Epithelioid mesotheliomas	0	1	0	4	2	0	2	0	1	4	1	0
Biphasic mesotheliomas	0	5	0	1	0	0	0	1	0	0	0	0
Sarcomatoid mesotheliomas	0	11	0	0	0	0	0	0	0	0	0	0
**Mesotheliomas, total**	**0**	**17** ***	**0**	**5**	**2**	**0**	**2**	**1**	**1**	**4**	**1**	**0**

Chi-square four-way test: * = p < 0.05; ** = p < 0.01; *** = p < 0.001

Mesotheliomas were present in 17 animals (34%) of group 2 (amosite asbestos positive control), in 5 animals (10%) of group 4 (fragmented Dialead K13D2U, high dose), in 2 animals (4%) of group 5 (CNT1-1, low dose), 2 animals (4%) in group 7 (USRN 20–30, low dose), in 1 animal (2%) in group 8 (USRN 20–30, high dose), in 1 animal (2%) in group 9 (OCSiAl Tuball, low dose), in 4 animals (8%) in group 10 (OCSiAl Tuball, high dose) and in 1 animal (2%) in group 11 (Nanocyl NC7000 long, low dose). Many mesotheliomas showed multifocal proliferation in the abdominal cavity (and tunica vaginalis) with infiltration of multiple organs and structures like diaphragm, omentum, mesentery, liver, spleen, pancreas, and intestine.

**Fig. 4:**
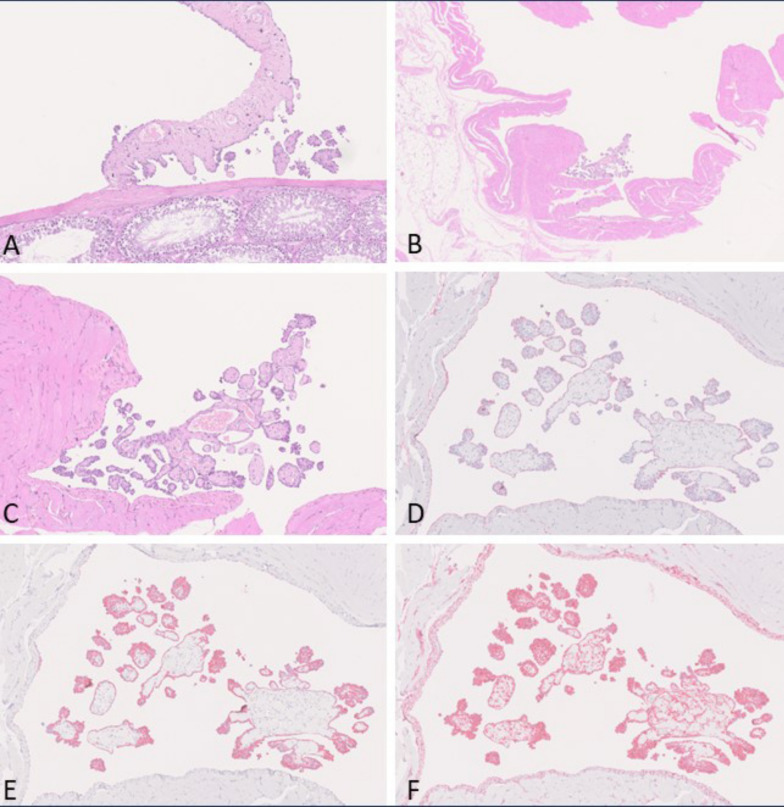
Early-stage epitheloid-type mesotheliomas observed in 2 animals of the OCSiAl Tuball high-dose group. **A**: H & E, small papillary proliferations of enlarged mesothelial cells on a poorly cellular, eosinophilic matrix arising in the tunica vaginalis in close association to the testis (10 ×). **B**: H & E, finger-shaped proliferations of mesothelial cells arranged on a poorly cellular, eosinophilic, vascularized stalk arising in the scrotum (5x). **C**: H & E, higher magnification of B (C: 10x). **D**: Anti-podoplanin immunohistochemistry of B revealing positive stained cytoplasm of many cells (10 ×). **E**: Anti-pan-cytokeratin immunohistochemistry of B showing many positive cells (10 ×). **F**: Anti-vimentin immunohistochemistry of B showing a strongly positive result (10 ×)

In 1 animal of group 4 (fragmented Dialead K13D2U, high dose), 1 animal of group 5 (CNT1-1, low dose) and 2 animals of group 10 (OCSiAl Tuball, high dose) mesotheliomas were localized just focally in the tunica vaginalis and therefore interpreted as early-stage epithelioid mesotheliomas. They appeared as finger-shaped proliferations of mesothelial cells, which were arranged on a poorly cellular, eosinophilic, vascularized stalk. These lesions were not present in any other examined localization. Immunohistochemically, early-stage epithelioid mesotheliomas were positive for vimentin, podoplanin and pan-cytokeratin (see Fig. 4).

In addition, mesotheliomas in group 2 (amosite asbestos positive control), group 4 (fragmented Dialead K13D2U, high dose), group 7 (USRN 20–30, low dose) and group 10 (OCSiAl Tuball, high dose) were exclusively located in the tunica vaginalis but had a multifocal appearance.

In animals of group 1 (medium control), group 3 (fragmented Dialead K13D2U, low dose), group 6 (CNT1-1, high dose) and group 12 (Nanocyl NC7000 long, high dose) no mesotheliomas were observed.

**Fig. 5:**
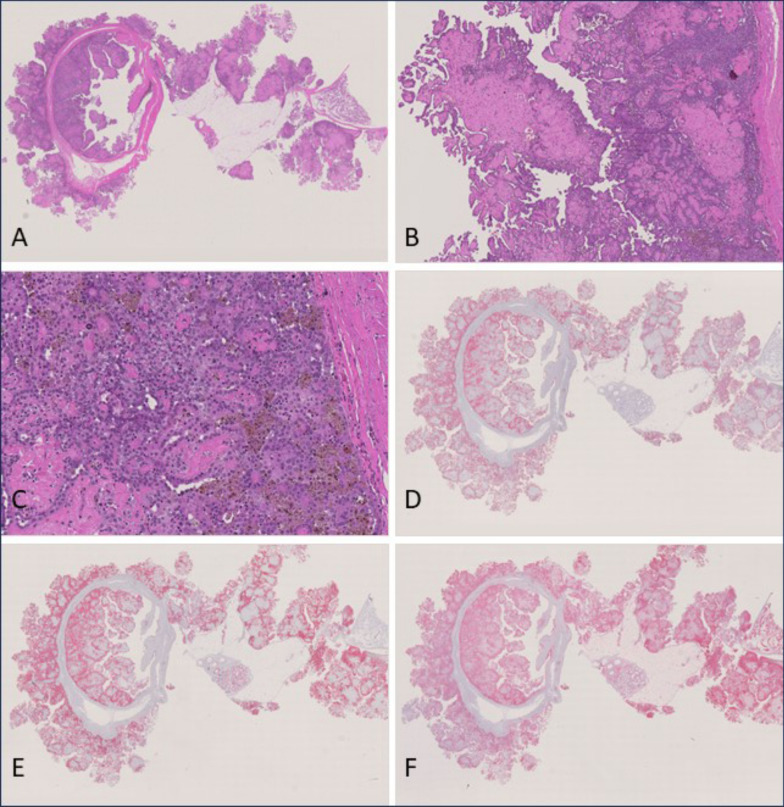
Epitheloid-type mesothelioma observed in the USRN 20–30 low-dose group. **A**: H & E, overview of tumor tissue invading the scrotum and tunica vaginalis (0.58 ×). **B**, **C**: H & E, higher magnification of A (B: 5 ×; C: 20x). **D**: Overview of anti-podoplanin immunohistochemistry revealing positively stained cytoplasm of many cells (0.62 ×). **E**: Overview of anti-pan-cytokeratin immunohistochemistry showing many positive cells (0.61 ×). **F**: Overview of anti-vimentin immunohistochemistry showing a strongly positive result (0.61 ×)

Regarding subtypes, most of the mesotheliomas were classified as epithelioid subtypes except mesotheliomas in group 2 (amosite asbestos positive control). Epithelioid mesotheliomas were characterized by superficial growth of rounded tumor cells on the serosal surface with frequent invasion of the underlying tissues. Tumor cells had rounded nuclei and displayed considerable pleomorphism. Typically, they formed papillary structures or fronds with a core consisting of poorly cellular homogenous to finely fibrillar eosinophilic vascularized stroma. Immunohistochemically, epithelioid mesotheliomas were positive for vimentin, podoplanin and pan-cytokeratin (see Fig. 5).

**Fig. 6:**
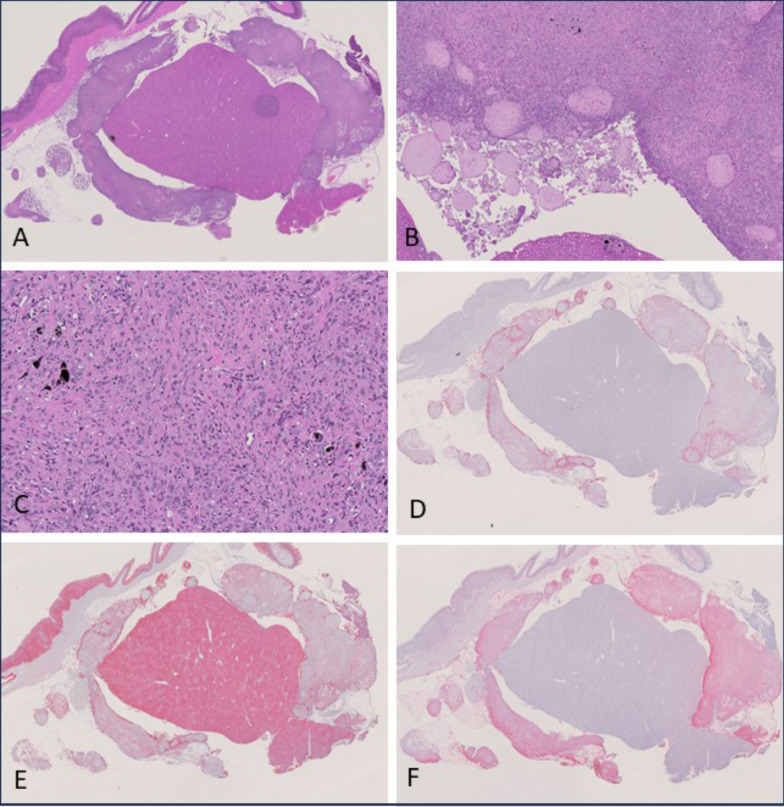
Biphasic-type mesothelioma observed in the USRN 20–30 high-dose group. A: H & E, overview of tumor tissue surrounding and partially invading the liver (0.67 ×). **B**, **C**: H & E, higher magnification of A (B: 5 ×; C: 20x). **D**: Overview of anti-podoplanin immunohistochemistry revealing positively stained cytoplasm of many cells (0.68 ×). **E**: Overview of anti-pan-cytokeratin immunohistochemistry showing many positive cells (0.67 ×). **F**: Overpower view of anti-vimentin immunohistochemistry showing a strongly positive result (0.69 ×)

Single mesotheliomas in group 4 (fragmented Dialead K13D2U, high dose) and group 8 (USRN 20–30, high dose) showed a biphasic subtype consisting of both epithelioid and sarcomatoid parts. Biphasic mesotheliomas showed positivity for vimentin, podoplanin and pan-cytokeratin via immunohistochemical staining (see Fig. 6).

**Fig. 7:**
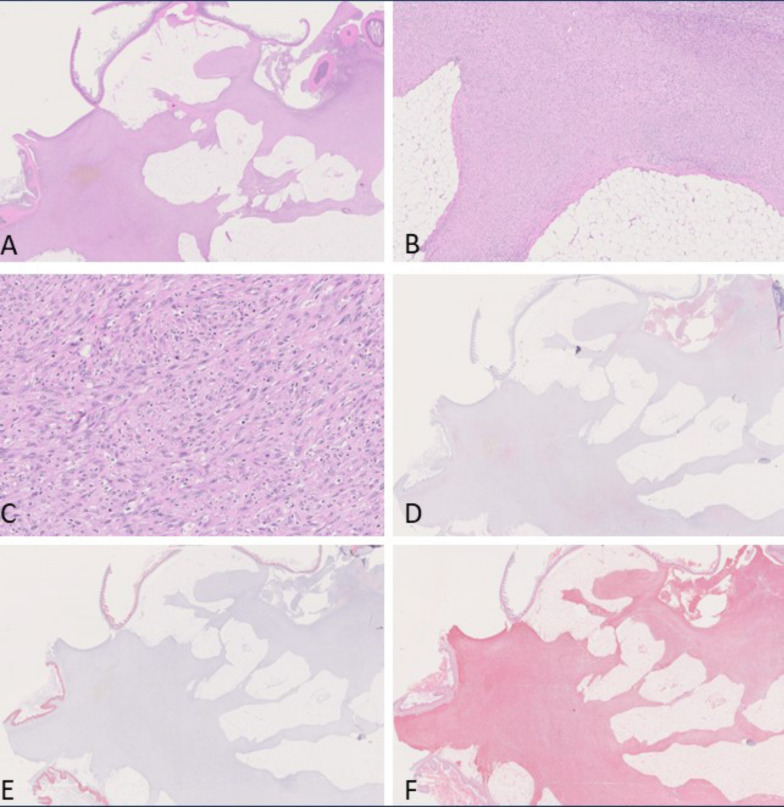
Sarcomatoid-type mesothelioma observed in the amosite asbestos control group. **A**: H & E, overview of tumor invading the abdominal adipose tissue (0.72 ×). **B**, **C**: H & E, higher magnification of A (B: 5 ×; C: 20x). **D**: Overview of anti-podoplanin immunohistochemistry revealing slightly positive stained cytoplasm of several cells (0.73 ×). **E**: Overview of anti-pan-cytokeratin immunohistochemistry showing negative tumor cells (0.69 ×). **F**: Overview of anti-vimentin immunohistochemistry showing a strongly positive result (0.71 ×)

The predominant mesothelioma subtype in group 2 (amosite asbestos positive control) was sarcomatoid mesothelioma (11/17), followed by biphasic mesothelioma (5/17) and 1 epithelioid mesothelioma. Sarcomatoid mesotheliomas showed a considerable grade of de-differentiation. They predominantly formed densely cellular sheaths around multiple organs of the abdominal cavity or infiltrated adjacent tissues. Tumor cells were spindle-shaped with elongated nuclei and accompanied by less amount of stroma. Immunohistochemically, they stained strongly positive for vimentin, but podoplanin and pan-cytokeratin were only positive in some tumor cells that were predominately located at the surface of the tumor (see Fig. 7).

Osseous metaplasia was frequently present independently from the mesothelioma subtype.

##### Mesothelial hyperplasia

After 3 months, mesothelial hyperplasia was observed within the mesentery of single animals of group 2 (amosite asbestos positive control) and group 3 (fragmented Dialead K13D2U, low dose) (supplementary Table S5).

**Fig. 8:**
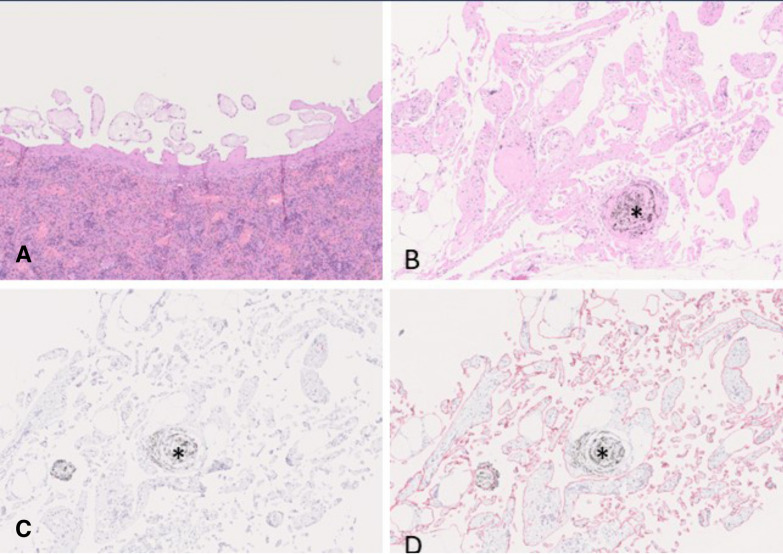
Mesothelial hyperplasia observed in the amosite asbestos control group (**A**) and the Nanocyl NC7000 long high dose group (**B**–**D**). A: H & E, papillary proliferations of flat mesothelial cells and a poorly cellular scantly stained extracellular matrix on the surface of the spleen (10 ×). **B**: H & E, frond-like proliferations of flat mesothelial cells arranged on a poorly cellular, eosinophilic extracellular matrix in the omentum in close association to a small granuloma (asterisk; 10x). **C**: Anti-Ki-67 immunohistochemistry of B revealing negative staining of mesothelial cells. There are a few positive immune cells present within the small granuloma (asterisk; 10x). **D**: Anti-podoplanin immunohistochemistry of B showing positive stained cytoplasm of mesothelial cells (10x)

After 24 months, many animals of the amosite asbestos group as well as group 4 (fragmented Dialead K13D2U, high dose), group 7 (USRN 20–30, low dose) and group 8 (USRN 20–30, high dose) showed partly papillary projections of the mesothelium on the surface of the spleen. Other affected locations were omentum, mesentery, tunica vaginalis, ligamentum falciforme, and diaphragm. Mesothelial cells in these areas were predominately flat and single layered and did not reveal any malignant features like pleomorphism or infiltrative growth. Furthermore, they did not display any proliferative activity in a Ki-67 immunohistochemical staining (see Fig. 8). Therefore, these lesions were interpreted as mesothelial hyperplasia (Table 7).

**Table 7:** Occurrence of mesothelial hyperplasia in protocol organs in the carcinogenicity study

	Medium control	Amosite asbestos	Dialead K13D2U Carbon fiber fragments		CNT1-1 MWCNT		USRN 20–30 MWCNT		OCSiAl Tuball SWCNT		Nanocyl NC7000 long MWCNT	
			Low	High	Low	High	Low	High	Low	High	Low	High
**Group**	**1**	**2**	**3**	**4**	**5**	**6**	**7**	**8**	**9**	**10**	**11**	**12**
Number of animals examined	(50)	(50)	(50)	(50)	(50)	(50)	(50)	(50)	(50)	(50)	(50)	(50)
Diaphragm	0	1	0	0	1	0	0	0	0	0	0	1
Falciform ligament	0	2	0	0	0	0	0	1	0	0	0	0
Spleen	2	**20** ***	2	**31** ***	3	**9** *	**14** **	**13** **	4	7	1	6
Mesentery	0	**7** *	0	0	0	1	0	0	1	0	0	0
Omentum	0	**9** **	1	0	2	0	0	2	2	1	1	0
Tunica vaginalis	0	0	2	2	3	3	0	0	0	0	1	1
Scrotum	0	3	0	0	0	1	0	0	1	0	0	1

Chi-square four-way test: * = p < 0.05; ** = p < 0.01; *** = p < 0.001

##### Other non-neoplastic MWCNT-induced histopathological findings in the abdominal cavity

Animals in the 3-month study had similar fiber-induced lesions as animals in the 24-month study, but the severity was generally lower (supplementary tables S6 and S7).

In all treated groups of the 24-month study, (multi)focal granulomatous inflammation, partly with granuloma formation, was observed in many animals within omentum, mesentery, diaphragm, liver (subserosa), ligamentum falciforme, spleen (subserosa), scrotum, testis (subserosa), pancreas (subserosa) and intestine (subserosa) (Table 8). Inflammatory cell populations predominantly consisted of (partly multinucleated) macrophages and lymphocytes as well as often neutrophilic granulocytes and small amounts of plasma cells. A reactive hypertrophy of mesothelial cells was frequently present in affected areas.

**Table 8:** Occurrence of granulomatous inflammation/granulomas in protocol organs in the carcinogenicity study

	Medium control	Amosite asbestos	Dialead K13D2U Carbon fiber fragments		CNT1-1 MWCNT		USRN 20–30 MWCNT		OCSiAl Tuball SWCNT		Nanocyl NC7000 long MWCNT	
			low	high	low	high	low	high	low	high	low	high
**Group**	**1**	**2**	**3**	**4**	**5**	**6**	**7**	**8**	**9**	**10**	**11**	**12**
Number of animals examined	(50)	(50)	(50)	(50)	(50)	(50)	(50)	(50)	(50)	(50)	(50)	(50)
Diaphragm, subserosa	0	**6** *	**37** ***	**46** ***	**14** ***	**32** ***	**4** *	**20** ***	0	**4** *	1	**24** ***
Diaphragm	0	0	4	**7** **	0	1	0	0	0	0	0	1
Liver, subserosa	0	**7** **	**19** ***	**45** ***	0	**30** ***	2	**17** ***	0	0	0	**22** ***
Liver	0	0	0	0	0	1	0	0	0	0	0	0
Falciform ligament	0	2	**17** ***	**39** ***	3	**24** ***	**4** *	**34** ***	0	0	1	**26** ***
Spleen, subserosa	0	2	**14** ***	**38** ***	3	**30** ***	1	**21** ***	1	3	**4** *	**22** ***
Pancreas, serosa	0	0	0	**19** ***	0	0	0	0	0	0	0	0
Pancreas	0	0	0	**8** **	0	0	0	0	0	0	0	0
Mesentery	0	**8** **	**45** ***	**47** ***	**25** ***	**34** ***	**6** *	**14** ***	2	**14** ***	3	**19** ***
Omentum	2	**14** **	**47** ***	**46** ***	**38** ***	**40** ***	**17** ***	**35** ***	3	**30** ***	**19** ***	**40** ***
Tunica vaginalis	0	0	**7** *	**18** ***	0	1	0	1	0	0	0	0
Intestine, subserosa	0	0	1	**15** ***	0	3	0	0	0	0	0	1
Intestine	0	0	2	2	0	1	0	0	0	0	0	2
Testes, subserosa	0	0	1	**20** ***	0	3	0	1	0	0	0	3
Scrotum	0	1	**10** ***	**38** ***	3	**8** **	0	5 *	0	2	0	**8** **

**Fig. 9:**
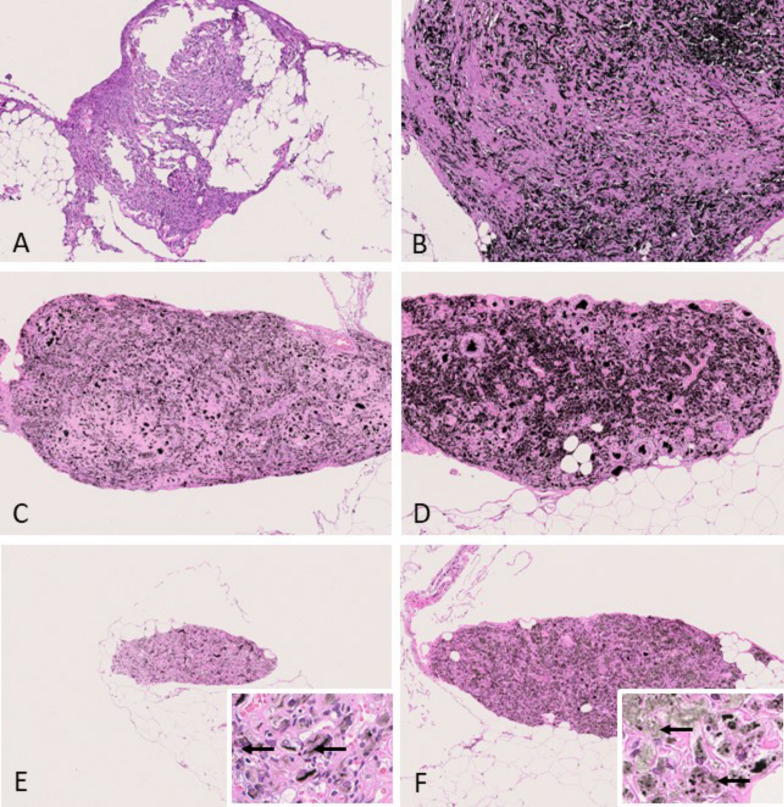
All fibers caused granulomas in the omentum. **A**: H & E, medium size granuloma in the omentum in the amosite asbestos positive control group (10 ×). **B**: H & E, large size granuloma in the omentum in the Dialead K13D2U high dose group (10 ×). **C**: H & E, medium size granuloma in the omentum in the CNT1-1 high dose group (10 ×). **D**: H & E, medium size granuloma in the omentum in the USRN 20–30 high dose group (10 ×). **E**: H & E, small size granuloma in the omentum in the OCSiAl Tuball high dose group (10 ×). Insert: higher magnification (40x) to illustrate fiber-laden macrophages (arrows). **F**: H & E, medium size granuloma in the omentum in the Nanocyl NC7000 long high dose group (10 ×). Insert: higher magnification (40x) to illustrate fiber-laden macrophages (arrows)

Granulomas often consisted of a central hyaline area with incorporated foreign material, surrounded by macrophages and lymphocytes as well as an outer layer with fibrocytes and collagen covered by mesothelium. In many animals, fiber-laden macrophages were present in these locations (see Fig. 9).

Incidences and severity grades of these lesions were highest in group 4 (fragmented Dialead K13D2U, high dose), followed by group 3 (fragmented Dialead K13D2U, low dose), group 6 (CNT1-1, high dose), group 8 (USRN 20–30, high dose), and group 12 (Nanocyl NC7000 long, high dose) (Table 8). Animals of the other treated groups, including amosite asbestos positive control group, revealed lower incidences and severity grades of granulomatous inflammation/ granulomas. In all treated groups (except group 9 [OCSiAl Tuball, low dose]) the number of animals with granulomatous inflammation/ granuloma in one or more of the protocol organs was statistically highly significant (p < 0.001) compared to the negative control group.

In some animals of group 3 (fragmented Dialead K13D2U, low dose), group 4 (fragmented Dialead K13D2U, high dose) as well as single animals of group 6 (CNT1-1, high dose) and group 12 (Nanocyl NC7000 long, high dose), granulomatous inflammation/ granulomas and fiber-laden macrophages were not only restricted to the sub-serosal areas of observed directly below the serosal surface of diaphragm, liver, pancreas, spleen, and intestine, but extended from the sub-serosal areas also deeper into these organs.

Furthermore, fibrosis was found in many animals of almost all groups in the spleen (subserosa), omentum and mesentery. Lesser numbers of animals showed fibrosis within the ligamentum falciforme, diaphragm (subserosa), liver (subserosa) and in only 1 animal fibrosis was present within the subserosal tissue of the pancreas (Table 9).

**Table 9:** Occurrence of fibrosis in protocol organs in the carcinogenicity study

	Medium control	Amosite asbestos	Dialead K13D2U Carbon fiber fragments		CNT1-1 MWCNT		USRN 20–30 MWCNT		OCSiAl Tuball SWCNT		Nanocyl NC7000 long MWCNT	
			low	high	low	high	low	high	low	high	low	high
**Group**	**1**	**2**	**3**	**4**	**5**	**6**	**7**	**8**	**9**	**10**	**11**	**12**
Number of animals examined	(50)	(50)	(50)	(50)	(50)	(50)	(50)	(50)	(50)	(50)	(50)	(50)
Diaphragm, subserosa	0	**11** ***	**9** **	2	0	2	1	1	**6** *	1	5	**9** **
Liver, subcapsular	1	11**	0	0	0	0	1	0	0	0	0	0
Ligamentum falciforme	1	7	3	**9** *	1	5	0	0	1	1	6	**14** ***
Spleen, subserosa	0	**24** ***	**20** ***	**38** ***	**4** *	**25** ***	**7** **	**14** ***	3	2	**9** **	**20** ***
Pancreas, cubserosa	0	**1**	0	0	0	0	0	0	0	0	0	0
Mesentery	0	**6** *	1	2	3	4	0	0	**13** ***	2	**9** **	**16** ***
Omentum	0	**12** ***	**15** ***	**18** ***	**15** ***	**19** ***	**6** *	**28** ***	**18** ***	**11** ***	**17** ***	**17** ***
Scrotum	0	3	1	0	0	0	0	1	0	0	0	2

##### Tumors other than mesothelioma in the abdominopelvic cavity

In addition to mesotheliomas, other malignant tumors like hepatocellular carcinomas, pancreatic islet-cell carcinomas, hemangiosarcomas of the spleen and mesenteric lymph nodes, and leiomyosarcomas and malignant schwannomas of the intestine were observed in the abdominal cavity (supplementary Table S8). Although these tumors were slightly more often observed in the treated groups, they were interpreted to occur independently of the treatment. Like the other tumors in different organs and tissues outside the abdominopelvic cavity (supplementary Table S9), their incidences corresponded to normal spontaneous background in age matched male Wistar rats [32, 33].

In some animals, malignant, often multicentric lymphomas were found. Especially in group 6 (CNT1-1, high dose), the number of animals with malignant lymphoma was relatively high (6/50). To distinguish different subtypes, immunohistochemical staining for CD3 (T-cell marker) and CD79α (B-cell marker) was performed. As tumor cells stained positive for CD3 (T-cell marker) or CD79α (B-cell marker) and in two cases for both antibodies, the lymphomas were interpreted to be spontaneous.

Other systemic tumors of the hematopoietic system such as histiocytic sarcoma and leukemia (not otherwise specified) occurred in single animals.

#### Image analysis of the 3-months study

**Fig. 10:**
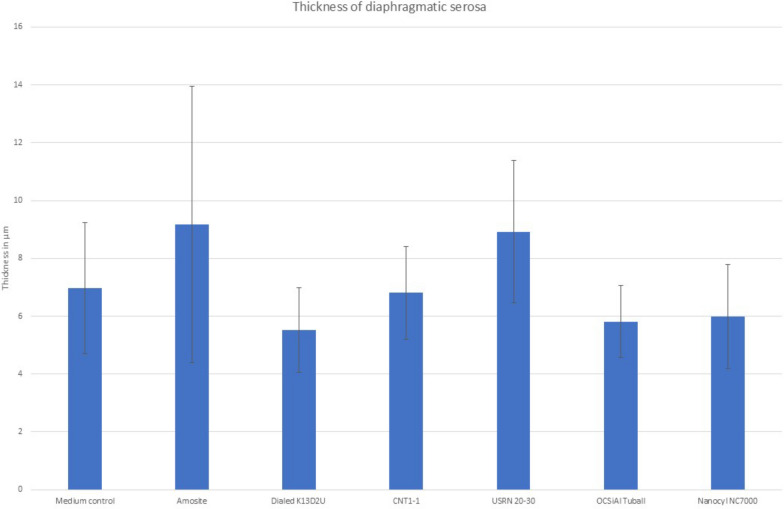
Diaphragmatic thickness measurement after 3 months of fiber application

The measurement of the thickness of the diaphragm showed a slight difference between the negative control group (7µm) and amosite (9.2 µm) or USRN 20–30 (8.9 µm) (Fig. 10, supplementary Table S10). However, the statistical analysis revealed no difference between the different groups (p = 0.531).

## Discussion

This long-term study examined effects of MWCNTs with diameters ranging from 10 to 30 nm. Additionally, a sample of single-walled carbon nanotubes (SWCNTs) exhibiting single fiber diameters significantly below 10 nm and showing a non-rigid morphology was included. However, individual SWCNT fibers can aggregate to form bundles that exhibit increased rigidity. In addition to the CNTs, this project also investigated the carcinogenic effect of fragments of a mesophase pitch-based carbon fiber.

Mesotheliomas occurred in all fiber groups, but not at all dosages. The thickest and therefore most rigid fibers in our study were present in the fragmented mesophase pitch-based carbon fibers of type Dialead K13D2U (GMD 1.2 µm), which resulted in 5 mesotheliomas in the high dose group but none in the low dose group. Different from PAN-based CF, pitch-based CF can show fiber splicing and splitting along the fiber axis [3, 29] resulting in the formation of fibrous fragments with WHO dimensions. To our knowledge, this is the first report investigating the carcinogenic potential of pitch-based CF fragments of respirable fiber morphology. Since we found 5 mesotheliomas in the group that got a dosage of 1 × 10^9^ WHO fibers, it would be of great interest to investigate a higher dosage of 5 × 10^9^ WHO fibers according to the EUR 18748 EN or to perform a long-time inhalations study to prove carcinogenic potential.

Among the investigated CNTs the thickest GMD of WHO-analog nanofibers was apparent in MWCNT USRN 20–30 (30 nm), so it was considered possibly rigid. This fiber type resulted in one biphasic mesothelioma in the peritoneal cavity of a high dose animal, which we interpret to be induced by the fibers. In the low dose group, one of two epitheloid mesotheliomas was found not only in the tunica vaginalis but also within the abdominal cavity. Since spontaneous mesotheliomas of the tunica vaginalis in rats usually do not spread to the abdominal cavity [34], we think that this tumor is also most likely fiber induced. Different to this, Varga and Szendi [26] tested a MWCNT material of 10–30 nm in diameter and 1–2 µm in length in groups of six Fischer 344 rats and did not observe any signs of mesothelioma after 12 months neither in the exposed nor in the control group. However, limitations of this study are the short time of investigation, as mesotheliomas occur after a duration > 12 months in asbestos control groups [35–37], the small group size of six animals only, and the lack of a positive control group. Moreover, the fibers used by these authors were substantially shorter compared to the fibers in our study (1–2 µm versus 6.85 µm). Several studies have highlighted that length plays an important role for the carcinogenic potential of fibers [38, 39]. It was hypothesized that longer fibers can get stuck in roughly 5 µm wide openings of the peritoneum, called stomata [40].

The MWCNT of type IFW CNT1-1 was also considered to be a possibly rigid fiber with its mean diameter of 20 nm. For this fiber, however only two well differentiated epitheloid mesotheliomas were observed in the low dose group, which were confined to the tunica vaginalis. Since such tumors are most likely of spontaneous origin, our results imply the diameter limit of non-carcinogenicity for MWCNTs is likely to be in the diameter range between 20 and 30 nm.

Accordingly, for the MWCNT Nanocyl NC7000 long research grade material with a GMD of about 11 nm only one most likely spontaneous epitheloid mesothelioma mainly located within the tunica vaginalis was observed in the low dose group. The GML of this fiber was 6.41 µm. With this, in our study fibers thinner than 20 nm diameter did not show asbestos-like effects even if they exceed the WHO fiber counting length limit of 5 µm.

However, it seems that fibers below a certain diameter might nonetheless be carcinogenic. We found (early) mesotheliomas in 5 animals treated with the OCSiAl Tuball SWCNT (apparent GMD about 7 nm, specified diameter 1.7 nm), which should be per se non-rigid but can form bundle aggregates of densely tangled fibers.

In contrast to our results, Nagai et al. [25] who injected a 15 nm diameter sized tangled MWCNT into the peritoneal cavity of Fischer 344/ Brown Norway F1 hybrids did not observe any mesothelioma development over the rat’s lifetime. However, the number of rats which were examined over a period longer than one year was small (6 animals). Also, Sakamoto et al. [41] examined the carcinogenic potential of straight and tangled MWCNTs by intraperitoneal injection into 15 male Fischer 344 rats per group and found that a maximum of one rat, which was administered tangled MWCNTs, developed a not-treatment-related mesothelioma after one year, while the straight MWCNTs induced mesotheliomas at very high incidences. In our opinion, this study also has some limitations like the short observation period of one year, the small number of investigated animals and the lacking determination of the fiber length. Regarding the non-carcinogenic effect of thin MWCNTs, the most cited study is that from [24]. These authors found up to 3/50, partly sarcomatoid, mesotheliomas in the abdominal cavity of MWCNT-treated Wistar rats and interpreted them as background lesions. However, published higher incidences of mesotheliomas in control Han Wistar rats (up to 6%) had combined mesothelioma with other tumors within the abdominal cavity such as sarcomas or carcinomas, only excluding tumors of the uterus [22]. As carcinomas and sarcomas of different origins often occur in the peritoneal cavity of rats, it can be assumed that the true incidence of mesothelioma in these studies is significantly lower. In contrast to rats of the Fischer 344 strain, spontaneous mesotheliomas of the peritoneum are very rare in Han Wistar rats [42], Okada et al., [43, 44], Carlus et al., [32, 33]. Also, in the previous study from Rittinghausen et al. [37], just 1 out of 50 control Wistar Han rats (2%) have been diagnosed with an epitheloid mesothelioma confined to the tunica vaginalis, being the area where spontaneous mesotheliomas usually emerge [32]. Although male rats have a slightly higher background incidence of mesotheliomas, female Wistar Han rats are not recommended to be used in such *i.p.* carcinogenicity studies due to their high rate of uterine adenocarcinomas, which spread within the abdominal cavity and, therefore, can interfere with mesothelioma diagnosis [45]. Other authors confirmed the toxicologic and carcinogenic potential of tangled MWCNTs [46, 47]. Therefore, additional long-term studies with a sufficient number of animals including a higher dose of 5 × 10^9^ WHO-analog nanofibers are needed to investigate the hazard posed by tangled-type SWCNTs/MWCNTs based on their collective morphology and rigidity. CNTs tangled in granular-shaped agglomerates could behave like PSLT (poor solubility, low toxicity) particles and show overload but not fiber-specific effects [48].

Overall, in the present study, the numbers of mesothelioma bearing animals were relatively small, especially in comparison with the study of Rittinghausen et al. [37], where mesothelioma incidences were high (up to 100%). We therefore intentionally have used lower concentrations of fibers (0.1 × 10^9^ and 1 × 10^9^ WHO fibers) in the current study, which could explain the lower mesothelioma incidences. The present study also intentionally explored CNT materials with smaller diameter (except CF fragments) than in the Rittinghausen study (7–30 nm versus 37–85 nm). As the flexural rigidity of a fiber scales with the fourth power of its diameter and proportionally to its bending modulus, the thinner a fiber, the more flexible it is, even if minor difference exists in the material property bending modulus. Long and flexible fibers can be internalized easier by macrophages [28, 49]. On the other hand, thin flexible fibers can align or entangle into (more or less densely packed) bundles or aggregates. If those exhibit collective fiber morphology, they can be longer and exhibit higher rigidity than the individual fibrils and therefore cannot be taken up so easily by macrophages compared to the constituting flexible single fibers. Moreover, thin MWCNTs have been reported to pierce mesothelial cells, resulting in a directly cytotoxic effect [25].

There are different possible modes of action of nanofibers leading to carcinogenesis. The bundle aggregates formed by the fibers are too long and collectively rigid to be successfully phagocytized by macrophages, leading to frustrated phagocytosis and inflammatory reactions [28, 50, 51]. If, due to biopersistence of the fibers, the inflammatory process persists over a long period, tumor formation can finally occur. Furthermore, telomere shortening induced by long MWCNTs is supposed to result in cell cycle arrest and apoptosis representing an important mechanism in MWCNT-induced inflammation and fibrosis as well as carcinogenesis (Alswady-Hoff et al., [52]). In vitro investigations have shown that long and straight MWCNTs can trigger premature cellular senescence in primary human mesothelial cells which can also be related to carcinogenesis (Reamon-Buettner et al., [53]). Recently, a retrospective study showed the influence of impaired molecular pathways that impact the genome, epigenome and translatome on the development of mesotheliomas by different MWCNTs and asbestos (Reamon-Buettner et al., [54]). Another possible mode of action is the interaction of thin individual SWCNT fibrils with the mitotic apparatus of mesothelial cells could result in altered cellular division and formation of tumors [55, 56].

Induced mesotheliomas are also supposed to emerge from the mesothelial lining of the tunica vaginalis [57]. The limited space within the scrotum results in greater mechanical stress of the mesothelial cells via friction, especially if the space is further limited by fiber-laden macrophages and granulomas, respectively. In our study, granulomas in the scrotal cavity were most often present in group 4 (fragmented Dialead K13D2U, high dose). This correlates with the higher incidence of mesotheliomas in this group. We found mesotheliomas in the abdominal cavity of 2/50 rats of group 4 and 1/50 rats of group 7, 8, 9, and 10, respectively, which are interpreted to be induced by the treatment with the fibers. In group 11, one rat also had an epitheloid mesothelioma, which was located not only in the tunica vaginalis, but also in the abdominal cavity. However, since the mesothelioma in the abdominal cavity of this animal was limited to a small area close to the spleen, we think this tumor has most likely arisen spontaneously. Mesotheliomas which were induced by asbestos fibers and CNTs are described to grow through the diaphragm to the pleural cavity [31]. These aggressive mesotheliomas are often of the sarcomatoid subtype, but also biphasic (and less often) epitheloid subtypes occur in induced mesotheliomas [31].

In the current study, we also classified mesotheliomas by morphologic and immunohistologic parameters into the epitheloid, biphasic and sarcomatoid subtype. This subclassification is of special interest because the biphasic and especially the sarcomatoid subtype occur in more advanced cases of mesotheliomas [58, 59]. In humans, this subclassification is also made by the 2021 World Health Organisation (WHO [60]) and incorporates additional architectural patterns (tubulopapillary, trabecular, adenomatoid, micropapillary, solid for the epitheloid subtype), cytological (and stromal) features (myxoid, lymphohistiocytoid, rhabdoid, transitional, desmoplastic, pleomorphic) as well as nuclear grading (low versus high) for the epitheloid subtype [61]. With this additional information, a prognostic significance (favorable versus unfavorable) is given.

In overall four animals (one in group 4, one in group 5 and two in group 10) we detected small, focal, neoplastic proliferations of the mesothelial outlining of the tunica vaginalis. They consisted of small fronds lined by enlarged, neoplastic mesothelial cells and a central stalk of vascularized hypocellular tissue. Since these proliferations were confined to a single location within the tunica vaginalis, we called them “early-stage mesotheliomas” to define them from other mesotheliomas which were multifocally located within the tunica vaginalis and/ or the peritoneal cavity. Similar observations were made by other authors [57–59] who described an early phase of mesothelioma as a small spherical nodule in the abdominal cavity which they called “spherical connective tissue rich (SCR)” epitheloid mesothelioma. In humans a so-called “localized malignant mesothelioma” is reported in the thoracic cavity, peritoneal cavity [62] and in the tunica vaginalis [63, 64]. This localized type of mesothelioma has a more favorable prognosis than diffuse malignant mesothelioma but could also become more malignant if no surgical removement is performed [62].

Papillary projections lined by flat mesothelial cells were most often detected on the surface or in close relation to the spleen. To prove the non-neoplastic character of these lesions we performed an immunohistochemistry using an antibody against the proliferation marker Ki-67. Since the mesothelial cells in these areas were negative for Ki-67 and had an overall inconspicuous morphology, the papillary projections were interpreted as mesothelial hyperplasia. In other locations (e.g. omentum, diaphragm) hyperplastic lesions were present as more diffuse thickening of the mesothelium. Atypical, and therefore pre-neoplastic, forms of mesothelial hyperplasia were not present in our study. Interestingly, mesothelial hyperplasia at the spleen was more often present in group 4 (fragmented Dialead K13D2U, high dose) than in the amosite asbestos control group, which correlated with the more advanced granulomatous inflammation in this group.

Like in other studies [59], the findings of granulomatous inflammation/granuloma, fibrosis and mesothelial hyperplasia were more often and more pronounced after a study duration of 24 months than after 3 months (interim sacrifice). This reflects an ongoing inflammatory process induced by the fiber administration which can finally result in mesothelioma development. However, the investigation at 3 months could not predict the study outcome after 24 months. Inflammation, oxidative stress, and tissue damage and repair play a crucial role in fiber-induced carcinogenesis, leading to DNA damage and mutations [65]. Besides, an immunosuppressive microenvironment caused by TGF-β-releasing M2-macrophages and other cells like monocytic Myeloid Derived Suppressor Cells (M-MDSC) in response to (intraperitoneal) CNTs seems to pioneer tumor development [59, 66, 67]. Interestingly, the different fibers differ in the severity grade of granulomas/ granulomatous inflammation. Size and numbers of granulomas were most advanced in groups 3 and 4 (fragmented Dialead K13D2U), followed by the MWCNT high dose groups 6 (CNT1-1), 8 (USRN 20–30) and 12 (Nanocyl NC7000 long). The smallest and most inconspicuous granulomas were present in the SWCNT groups 9 and 10 (OCSiAl Tuball). Therefore, other pathogenic processes such as inflammation seem to drive mesotheliomagenesis at least in the SWCNT group, as was mentioned before.

## Conclusion

For fragments derived from the carbon fiber Dialead K13D2U, there is a weak carcinogenic potential in the high dosage (group 4). The predominantly very good epithelioid differentiation and the low number of mesotheliomas indicate a weak and very weak carcinogenic potential for the rat in the intraperitoneal test for the for the SWCNT fiber OCSiAl Tuball and MWCNT fiber USRN 20–30, respectively. There is no clear carcinogenic potential for the fibers Nanocyl NC7000 long and CNT1-1. The limit, below which no asbestos-like effect is to be expected for MWCNTs, is therefore likely to be in the diameter range between 20 and 30 nm. SWCNTs also bear a carcinogenic potential, but the underlying mechanisms have to be further evaluated.

## Supplementary Information

Supplementary Material 1.

Supplementary Material 2.

Supplementary Material 3.

Supplementary Material 4.

Supplementary Material 5.

Supplementary Material 6.

Supplementary Material 7.

Supplementary Material 8.

Supplementary Material 9.

Supplementary Material 10.

Supplementary Material 11.

## Data Availability

Data is provided within the manuscript or supplementary information files.

## Abbreviations

µm: Micrometer
Agglom.: Agglomerate
CF: Carbon fiber
CNT: Carbon nanotube
D: Diameter
DM: Dispersion medium
F_WHO_: WHO fibers or WHO-analog nanofibers
GMD: Geometric mean diameter
GML: Geometric mean length
*i.p.*: Intraperitoneal
L: Length
mg: Milligram
min: Minute(s)
MWCNT: Multi-walled carbon nanotube
nm: Nanometer
Part.: Particle
PSD: Particle size distribution
SD: Standard deviation
SPF: Specific-pathogen-free
SWCNT: Single-walled carbon nanotube
TRGS: Technical Rules for Hazardous Substances
WHO: World Health Organization
