# Supplementary figures and images for: Impact of carbon-based fibers morphologies on their carcinogenic potential
Source: Part Fibre Toxicol. 2026 Feb 7;23:7. doi: 10.1186/s12989-026-00663-y (PMC12931056; doi:10.1186/s12989-026-00663-y)

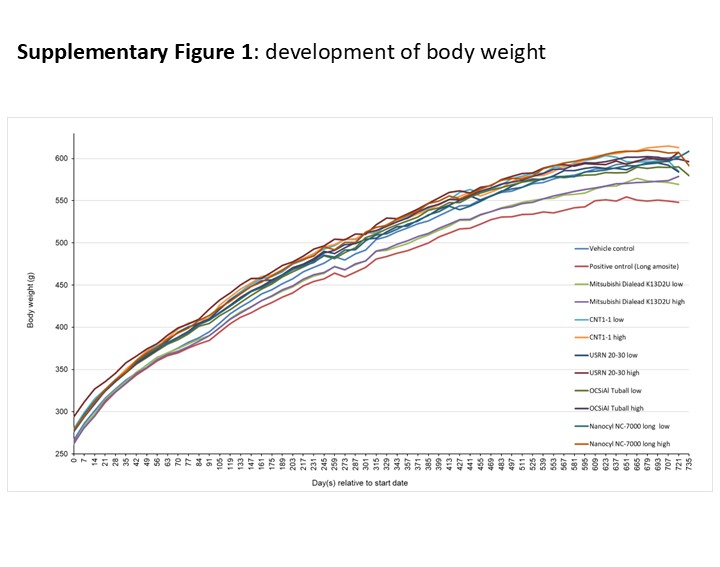

Supplement: Supplementary file 1 — Supplementary Material 1. [file 12989_2026_663_MOESM1_ESM.jpg]
