# Supplementary material for: Impact of carbon-based fibers morphologies on their carcinogenic potential
Source: Part Fibre Toxicol. 2026 Feb 7;23:7. doi: 10.1186/s12989-026-00663-y (PMC12931056; doi:10.1186/s12989-026-00663-y)
Supplement: Supplementary file 2 — Supplementary Material 2. [file 12989_2026_663_MOESM2_ESM.docx]

**Supplementary table 1** Particle size distribution of the test materials in the final preliminary test batch

|  | | Material class 1 | Material class 2 | Material class 3 | Material class 4 | Material class 5 | Positive control |
| --- | --- | --- | --- | --- | --- | --- | --- |
|  |  | SWCNT | MWCNT | MWCNT | MWCNT | Carbon fiber fragment | Asbestos |
|  |  | OCSiAl  Tuball | Nanocyl  NC-7000 | IFW  CNT1_1 | USRN  20-30 nm | Mitsubishi  Dialed K13D2U | Long Amosite |
| Dispersion | Ultrasound duration [min] | 2 x 5 | 1 | 1 | 2 | 18 h magnetic stirrer, US bath 15 min, 2 min US tip (100 %) | 2 x 5 |
|  | Ultrasound amplitude [%] | 100 | 100 | 30  (short 100) | 30  (short 100) |  | 100 |
| Concentration | Number  F_WHO_/mg material | 107.9 x 10^9^ | 14.4 x 10^9^ | 3.1 x 10^9^ | 1.5 x 10^9^ | 0.032 x 10^9*^ | 0.155 x 10^9^ |
| Relative percentage | F_WHO_ | 6.3% | 3.6% | 3.92% | 1.7% | 26.7%* | 59.5% |
|  | 3.5 ≤ L ≤ 5 µm | 6.8% | 0.8% | 3.61% | 0.0% | 17.8% | 11.3% |
|  | < 3.5 µm | 86.9% | 93.6% | 88.20% | 97.1% | 17.5% | 28.1% |
|  | L/D < 3  (Agglom./Part.) | 0.0% | 1.2% | 4.27% | 1.2% | 36.1% | 1.0% |
| PSD  F_WHO_ | **GML (µm)** | **6.72** | **6.41** | **7.35** | **6.85** | **7.26** | **12.63** |
|  | SD | 1.26 | 1.22 | 1.38 | 1.30 | 1.30 | 1.98 |
|  | **GMD (µm)** | **0.007** | **0.011** | **0.020** | **0.030** | **1.20** | **0.37** |
|  | SD | 1.594 | 1.566 | 1.489 | 1.57 | 1.547 | 1.71 |
| PSD Fibers  3.5 < L < 5 µm | GML (µm) | 3.99 | 4.40 | 4.04 | 0 | 4.32 | 4.16 |
|  | SD | 1.06 | 1.00 | 1.11 | 0 | 1.12 | 1.11 |
|  | GMD (µm) | 0.013 | 0.020 | 0.024 | 0 | 0.834 | 0.27 |
|  | SD | 1.386 | 1.000 | 1.185 | 0 | 1.358 | 1.71 |
| PSD  L/D > 3  (all fibers) | GML (µm) | 1.70 | 0.94 | 1.46 | 1.14 | 4.81 | 6.72 |
|  | SD | 1.92 | 2.22 | 1.90 | 1.72 | 1.66 | 2.64 |
|  | GMD (µm) | 0.007 | 0.010 | 0.020 | 0.022 | 0.864 | 0.31 |
|  | Std | 1.696 | 1.574 | 1.469 | 1.450 | 1.824 | 1.79 |

* Note: Material contains also fibers longer than 5 µm and with a diameter ≥3 µm to a minor extent. The value of 0.035 x 10^9^ fibers was initially used to calculate weight in the suspensions prepared for the study. The values in this table (e.g. 0.032 x 10^9^ WHO fibers or a percentage of 26.7%) are the corrected values.

F_WHO_ refers to WHO-analog nanofibers in the case of CNTs and to WHO fibers in the case of carbon fibers and the positive control.

Short means few sections.
US: ultrasound; Agglom: agglomerates; Part.: particles; L: length, D: diameter; PSD: particle size distribution; GML: geometric length; GMD: geometric diameter; SD: standard deviation.
