# Supplementary material for: Impact of carbon-based fibers morphologies on their carcinogenic potential
Source: Part Fibre Toxicol. 2026 Feb 7;23:7. doi: 10.1186/s12989-026-00663-y (PMC12931056; doi:10.1186/s12989-026-00663-y)
Supplement: Supplementary file 3 — Supplementary Material 3. [file 12989_2026_663_MOESM3_ESM.docx]

**Supplementary table 2.1** Particle size distribution OCSiAl Tuball (main study)

|  | | low | high |
| --- | --- | --- | --- |
| Concentration | Target concentration/animal | 0.1x 10^9^ | 1 x 10^9^ |
|  | Number F_WHO_/mg material | 83.8 x 10^9^ | 113.8 x 10^9^ |
|  | Actual concentration/animal | **0.08 x 10^9^** | **1.06 x 10^9^** |
| Rel. percentage | WHO | 13.51% | 8.27% |
|  | 3.5 <= L <= 5 µm | 10.86% | 2.12% |
|  | < 3.5 µm | 75.52% | 89.57% |
|  | L/D < 3 (Agglom./Part.) | 0.11% | 0.03% |
| PSD  F_WHO_ | **GML (µm)** | 6.89 | 6.76 |
|  | Std | 1.27 | 1.32 |
|  | **GMD (µm)** | 0.008 | 0.007 |
|  | Std | 1.526 | 1.505 |
| PSD  Fibers 3.5 < L < 5 µm | Geom. mean length (µm) | 4.29 | 4.17 |
|  | Std | 1.1 | 1.12 |
|  | Geom. mean diameter (µm) | 0.007 | 0.009 |
|  | Std | 1.444 | 1.433 |
| PSD  L/D > 3 (All fibers) | Geom. mean length (µm) | 1.65 | 1.27 |
|  | Std | 2.64 | 2.43 |
|  | Geom. mean diameter (µm) | 0.007 | 0.007 |
|  | Std | 1.528 | 1.528 |

Agglom: agglomerates; Part.: particles; L: length, D: diameter; PSD: particle size distribution; GML: geometric length; GMD: geometric diameter; SD: standard deviation.

**Supplementary table 2.2** Particle size distribution Nanocyl NC-7000 (main study)

|  | | low | high (1)* | high (2)* | high (3)* | high (4)* | high (5)* |
| --- | --- | --- | --- | --- | --- | --- | --- |
| Concentration | Target concentration/animal | 0.1 x 10^9^ | 1 x 10^9^ | 1 x 10^9^ | 1 x 10^9^ | 1 x 10^9^ | 1 x 10^9^ |
|  | Number F_WHO_/mg material | 15.2 x 10^9^ | 15.9 x 10^9^ | 13.1 x 10^9^ | 12.9 x 10^9^ | 12.4 x 10^9^ | 10.8 x 10^9^ |
|  | Actual concentration/animal | **0.11 x 10^9^** | **1.15 x 10^9^** | **0.94 x 10^9^** | **0.91 x 10^9^** | **0.83 x 10^9^** | **0.78 x 10^9^** |
| Rel. percentage | WHO | 5.31% | 3.56% | 3.24% | 3.00% | 2.53% | 3.27% |
|  | 3.5 <= L <= 5 µm | 10.25% | 6.71% | 9.15% | 3.75% | 6.60% | 7.29% |
|  | < 3.5 µm | 83.07% | 88.79% | 86.88% | 92.19% | 90.04% | 88.55% |
|  | L/D < 3 (Agglom./Part.) | 1.36% | 0.94% | 0.73% | 1.06% | 0.83% | 0.90% |
| PSD  F_WHO_ | GML (µm) | **6.53** | **6.24** | **6.50** | **6.39** | **6.35** | **6.22** |
|  | Std | 1.27 | 1.18 | 1.28 | 1.24 | 1.23 | 1.24 |
|  | GMD (µm) | **0.012** | **0.010** | **0.011** | **0.011** | **0.010** | **0.010** |
|  | Std | 1.431 | 1.515 | 1.331 | 1.378 | 1.369 | 1.35 |
| PSD  Fibers 3.5 < L < 5 µm | Geom. mean length (µm) | 4.08 | 4.00 | 3.97 | 4.02 | 4.12 | 3.99 |
|  | Std | 1.11 | 1.11 | 1.09 | 1.12 | 1.10 | 1.14 |
|  | Geom. mean diameter (µm) | 0.013 | 0.009 | 0.01 | 0.006 | 0.009 | 0.009 |
|  | Std | 1.539 | 1.286 | 1.289 | 1.274 | 1.296 | 1.342 |
| PSD  L/D > 3 (All fibers) | Geom. mean length (µm) | 1.35 | 1.28 | 1.37 | 1.09 | 1.24 | 1.24 |
|  | Std | 2.22 | 2.17 | 1.99 | 2.10 | 2.16 | 2.16 |
|  | Geom. mean diameter (µm) | 0.011 | 0.009 | 0.010 | 0.007 | 0.009 | 0.009 |
|  | Std | 1.494 | 1.526 | 1.374 | 1.449 | 1.396 | 1.443 |

* the preparation of the high dose fiber suspension was performed in five batches

Agglom: agglomerates; Part.: particles; L: length, D: diameter; PSD: particle size distribution; GML: geometric length; GMD: geometric diameter; SD: standard deviation.

**Supplementary table 2.3** Particle size distribution IFW CNT1_1 (main study)

|  | | low | high (1)* | high (2)* | high (3)* | high (4)* | high (5)* |
| --- | --- | --- | --- | --- | --- | --- | --- |
| Concentration | Target concentration/ animal | 0.1 x 10^9^ | 1 x 10^9^ | 1 x 10^9^ | 1 x 10^9^ | 1 x 10^9^ | 1 x 10^9^ |
|  | Number  F_WHO_/mg material | 2.4 x 10^9^ | 2.3 x 10^9^ | 2.5 x 10^9^ | 2.4 x 10^9^ | 2.4 x 10^9^ | 2.7 x 10^9^ |
|  | **Actual concentration/ animal** | **0.08 x 10^9^** | **0.74 x 10^9^** | **0.81 x 10^9^** | **0.77 x 10^9^** | **0.77 x 10^9^** | **0.85 x 10^9^** |
| Rel. percentage | F_WHO_ | 4.00% | 2.86% | 4.41% | 3.05% | 3.11% | 3.93% |
|  | 3,5 ≤ L <= 5 µm | 3.75% | 3.81% | 6.09% | 3.94% | 5.00% | 3.82% |
|  | < 3,5 µm | 87.18% | 90.88% | 85.32% | 90.15% | 89.46% | 89.10% |
|  | L/D < 3 (Agglom./Part.) | 5.07% | 2.46% | 4.18% | 2.86% | 2.43% | 3.15% |
| PSD  F_WHO_ | **GML (µm)** | **7.42** | **7.30** | **7.17** | **7.38** | **7.25** | **7.71** |
|  | SD | 1.35 | 1.37 | 1.37 | 1.39 | 1.4 | 1.42 |
|  | **GMD (µm)** | **0.020** | **0.019** | **0.020** | **0.019** | **0.019** | **0.018** |
|  | SD | 1.483 | 1.417 | 1.445 | 1.482 | 1.457 | 1.435 |
| PSD  Fibers 3.5 < L < 5 µm | GML (µm) | 4.02 | 4.13 | 3.99 | 4.15 | 4.49 | 4.42 |
|  | SD | 1.05 | 1.09 | 1.14 | 1.06 | 1.08 | 1.11 |
|  | GMD (µm) | 0.016 | 0.017 | 0.022 | 0.018 | 0.018 | 0.018 |
|  | SD | 1.582 | 1.382 | 1.724 | 1.417 | 1.335 | 1.555 |
| PSD  L/D > 3 (all fibers) | GML (µm) | 1.25 | 1.18 | 1.26 | 1.09 | 1.1 | 1.16 |
|  | SD | 2.05 | 2.04 | 2.02 | 2.01 | 2.17 | 2.04 |
|  | GMD (µm) | 0.018 | 0.017 | 0.018 | 0.018 | 0.015 | 0.017 |
|  | SD | 1.464 | 1.451 | 1.464 | 1.449 | 1.469 | 1.435 |

* the preparation of the high dose fiber suspension was performed in five batches

Agglom: agglomerates; Part.: particles; L: length, D: diameter; PSD: particle size distribution; GML: geometric length; GMD: geometric diameter; SD: standard deviation.

**Supplementary table 2.4** Particle size distribution USRN20-30 (main study)

|  | | low | high (1)* | high (2)* | high (3)* | high (4)* | high (5)* |
| --- | --- | --- | --- | --- | --- | --- | --- |
| Concentration | Target concentration/ animal | 0.1 x 10^9^ | 1 x 10^9^ | 1 x 10^9^ | 1 x 10^9^ | 1 x 10^9^ | 1 x 10^9^ |
|  | Number  F_WHO_/mg material | 1.5x 10^9^ | 1.2 x 10^9^ | 1.4 x 10^9^ | 1.2 x 10^9^ | 1.2 x 10^9^ | 1.2 x 10^9^ |
|  | **Actual concentration/ animal** | **0.10 x 10^9^** | **0.81 x 10^9^** | **0.94 x 10^9^** | **0.80 x 10^9^** | **0.79 x 10^9^** | **0.78 x 10^9^** |
| Rel. percentage | F_WHO_ | 3.10% | 1.54% | 2.30% | 1.96% | 1.46% | 1.50% |
|  | 3,5 ≤ L ≤ 5 µm | 5.21% | 3.36% | 4.16% | 2.77% | 1.40% | 5.36% |
|  | < 3,5 µm | 90.50% | 94.93% | 93.37% | 95.17% | 97.09% | 93.13% |
|  | L/D < 3 (Agglom./Part.) | 1.18% | 0.17% | 0.17% | 0.10% | 0.05% | 0.00% |
| PSD  F_WHO_ | **GML (µm)** | **6.72** | **6.66** | **6.79** | **6.84** | **6.72** | **6.84** |
|  | SD | 1.32 | 1.3 | 1.35 | 1.35 | 1.28 | 1.32 |
|  | **GMD (µm)** | **0.027** | **0.029** | **0.030** | **0.029** | **0.03** | **0.029** |
|  | SD | 1.53 | 1.087 | 1.127 | 1.09 | 1.101 | 1.094 |
| PSD  Fibers 3.5 < L < 5 µm | GML (µm) | 4.00 | 4.07 | 3.82 | 4.21 | 3.86 | 4.06 |
|  | SD | 1.13 | 1.11 | 1.09 | 1.08 | 1.06 | 1.05 |
|  | GMD (µm) | 0.039 | 0.029 | 0.034 | 0.028 | 0.031 | 0.032 |
|  | SD | 1.248 | 1.036 | 1.128 | 1.018 | 1.000 | 1.115 |
| PSD  L/D > 3 (all fibers) | GML (µm) | 1.3 | 1.11 | 1.15 | 1 | 0.93 | 1.05 |
|  | SD | 2.05 | 1.95 | 1.9 | 1.92 | 2.07 | 1.92 |
|  | GMD (µm) | 0.025 | 0.023 | 0.025 | 0.026 | 0.025 | 0.025 |
|  | SD | 1.485 | 1.352 | 1.395 | 1.268 | 1.312 | 1.322 |

* the preparation of the high dose fiber suspension was performed in five batches

Agglom: agglomerates; Part.: particles; L: length, D: diameter; PSD: particle size distribution; GML: geometric length; GMD: geometric diameter; SD: standard deviation.

**Supplementary table 2.5** Particle size distribution of carbon fiber Mitsubishi Dialed K13D2U (main study)

|  | | low (1)* | low (2)* | high (1_1)** | high (1_2)** | high (2_1)** | high (2_2)** |
| --- | --- | --- | --- | --- | --- | --- | --- |
| Concentration | Target concentration/animal | 0.05 x 10^9^ | 0.05 x 10^9^ | 0.5 x 10^9^ | 0.5 x 10^9^ | 0.5 x 10^9^ | 0.5 x 10^9^ |
|  | Number  F_WHO_/mg material | 0.029 x 10^9^ | 0.027 x 10^9^ | 0.031 x 10^9^ | 0.036 x 10^9^ | 0.027 x 10^9^ | 0.032 x 10^9^ |
|  | **Actual concentration/animal** | **0.04 x 10^9^** | **0.04 x 10^9^** | **0.45 x 10^9^** | **0.52 x 10^9^** | **0.39 x 10^9^** | **0.46 x 10^9^** |
| Rel. percentage | Fasern L > 5µm | 36.5% | 43.8% | 35.7% | 42.3% | 30.6% | 32.6% |
|  | F_WHO_ | 30.1% | 36.6% | 31.2% | 38.1% | 27.0% | 28.5% |
|  | Fasern L > 5µm,  D ≥ 3 µm | 6.4% | 7.1% | 4.4% | 4.2% | 3.6% | 4.1% |
|  | 3,5 ≤ L ≤ 5 µm | 15.3% | 19.8% | 19.4% | 18.3% | 17.7% | 17.5% |
|  | < 3,5 µm | 14.2% | 9.9% | 18.5% | 14.0% | 11.3% | 13.3% |
|  | L/D < 3 (Agglom./Part.) | 34.0% | 26.6% | 26.4% | 25.4% | 40.4% | 36.6% |
| PSD  F_WHO_ | **GML (µm)** | **7.13** | **7.37** | **7.06** | **7.38** | **7.08** | **7.09** |
|  | SD | 1.32 | 1.31 | 1.30 | 1.28 | 1.31 | 1.30 |
|  | **GMD (µm)** | **1.245** | **1.269** | **1.276** | **1.337** | **1.271** | **1.242** |
|  | SD | 1.582 | 1.465 | 1.456 | 1.499 | 1.564 | 1.532 |
| PSD  Fibers 3.5 < L < 5 µm | GML (µm) | 4.16 | 4.25 | 4.26 | 4.28 | 4.20 | 4.18 |
|  | SD | 1.11 | 1,.2 | 1.12 | 1.11 | 1.12 | 1.11 |
|  | GMD (µm) | 0.827 | 0.818 | 0.862 | 0.836 | 0.875 | 0.862 |
|  | SD | 1.384 | 1.64 | 1.468 | 1.478 | 1.471 | 1.429 |
| PSD  L/D > 3 (all fibers) | GML (µm) | 5.20 | 5.71 | 4.88 | 5.38 | 5.09 | 5.04 |
|  | SD | 1.68 | 1.60 | 1.61 | 1.58 | 1.55 | 1.58 |
|  | GMD (µm) | 1.007 | 1.064 | 0.964 | 1.045 | 1.025 | 0.984 |
|  | SD | 1.769 | 1.75 | 1.647 | 1.661 | 1.63 | 1.631 |

* The target concentration refers to a single administration. The carbon fiber was administered twice per animal on two consecutive days; ** the preparation of the high dose fiber suspension was performed in five batches and was administered twice per animal on two consecutive days

Agglom: agglomerates; Part.: particles; L: length, D: diameter; PSD: particle size distribution; GML: geometric length; GMD: geometric diameter; SD: standard deviation.

**Supplementary table 2.5** (continued): Particle size distribution of carbon fiber Mitsubishi Dialed K13D2U (main study)

|  | | high (3_1)** | high (3_2)** | high (4_1)** | high (4_2)** | high (5_1)** | high (5_2)** |
| --- | --- | --- | --- | --- | --- | --- | --- |
| Concentration | Target concentration/animal * | 0.5 x 10^9^ | 0.5 x 10^9^ | 0.5 x 10^9^ | 0.5x10^9^ | 0.5 x 10^9^ | 0.5 x 10^9^ |
|  | Number  F_WHO_/mg material | 0.027 x 10^9^ | 0.027 x 10^9^ | 0.028 x 10^9^ | 0.023x10^9^ | 0.027 x 10^9^ | 0.029 x 10^9^ |
|  | **Actual concentration/animal** | **0.39 x 10^9^** | **0.39 x 10^9^** | **0.41x10^9^** | **0.33 x 10^9^** | **0.39 x 10^9^** | **0.41 x 10^9^** |
| Rel. percentage | Fibers L> 5 µm | 32.4% | 38.7% | 36.5% | 37.0% | 33.3% | 32.2% |
|  | F_WHO_ | 28.2% | 34.2% | 32.2% | 31.6% | 29.3% | 28.2% |
|  | Fibers L> 5 µm,  D ≥ 3 µm | 4.2% | 4.6% | 4.3% | 5.4% | 4.0% | 3.9% |
|  | 3,5 ≤ L ≤ 5 µm | 18.5% | 14.6% | 19.5% | 17.7% | 18.7% | 14.5% |
|  | < 3,5 µm | 9.5% | 9.0% | 11.4% | 19.0% | 12.1% | 17.1% |
|  | L/D < 3 (Agglom./Part.) | 39.7% | 37.6% | 32.6% | 26.3% | 35.8% | 36.2% |
| PSD  F_WHO_ | **GML (µm)** | **7.21** | **7.45** | **7.30** | **7.51** | **7.19** | **6.78** |
|  | SD | 1.33 | 1.32 | 1.31 | 1.35 | 1.34 | 1.29 |
|  | **GMD (µm)** | **1.305** | **1.360** | **1.385** | **1.318** | **1.280** | **1.217** |
|  | SD | 1.518 | 1.536 | 1.458 | 1.519 | 1.539 | 1.589 |
| PSD  Fibers 3.5 < L < 5 µm | GML (µm) | 4.18 | 4.25 | 4.32 | 4.27 | 4.27 | 4.23 |
|  | SD | 1.11 | 1.10 | 1.12 | 1.11 | 1.12 | 1.12 |
|  | GMD (µm) | 0.855 | 0.846 | 0.916 | 0.868 | 0.851 | 0.788 |
|  | SD | 1.395 | 1.433 | 1.371 | 1.52 | 1.59 | 1.527 |
| PSD  L/D > 3 (all fibers) | GML (µm) | 5.32 | 5.74 | 5.46 | 5.07 | 5.17 | 4.79 |
|  | SD | 1.55 | 1.57 | 1.56 | 1.64 | 1.58 | 1.62 |
|  | GMD (µm) | 1.04 | 1.118 | 1.118 | 0.961 | 0.983 | 0904 |
|  | SD | 1.636 | 1.702 | 1.593 | 1.761 | 1.743 | 1.807 |

* The target concentration refers to a single administration. The carbon fiber was administered twice per animal on two consecutive days; ** the preparation of the high dose fiber suspension was performed in five batches and was administered twice per animal on two consecutive days

Agglom: agglomerates; Part.: particles; L: length, D: diameter; PSD: particle size distribution; GML: geometric length; GMD: geometric diameter; SD: standard deviation.

**Supplementary table 2.6** Particle size distribution of long amosite (main study)

|  | |  |
| --- | --- | --- |
| Concentration | Target concentration/animal | 0.1 x 10^9^ |
|  | Number  F_WHO_/mg material | 0.14 x 10^9^ |
|  | **Actual concentration/animal** | **0.09 x 10^9^** |
| Rel. percentage | F_WHO_ | 57.80% |
|  | 3,5 ≤ L ≤ 5 µm | 14.56% |
|  | < 3,5 µm | 26.20% |
|  | L/D < 3 (Agglom./Part.) | 1.44% |
| PSD  F_WHO_ | **GML (µm)** | **12.93** |
|  | SD | 1.96 |
|  | **GMD (µm)** | **0.371** |
|  | SD | 1.734 |
| PSD  Fibers 3.5 < L < 5 µm | GML (µm) | 4.11 |
|  | SD | 1.12 |
|  | GMD (µm) | 0.311 |
|  | SD | 1.885 |
| PSD  L/D > 3 (all fibers) | GML (µm) | 6.77 |
|  | SD | 2.61 |
|  | GMD (µm) | 0.322 |
|  | SD | 1.809 |

Agglom: agglomerates; Part.: particles; L: length, D: diameter; PSD: particle size distribution; GML: geometric length; GMD: geometric diameter; SD: standard deviation.
