# Supplementary material for: Impact of carbon-based fibers morphologies on their carcinogenic potential
Source: Part Fibre Toxicol. 2026 Feb 7;23:7. doi: 10.1186/s12989-026-00663-y (PMC12931056; doi:10.1186/s12989-026-00663-y)
Supplement: Supplementary file 6 — Supplementary Material 6. [file 12989_2026_663_MOESM6_ESM.docx]

**Supplementary Table 5**: Occurrence of mesothelial hyperplasia in protocol organs 3 months after intraperitoneal injection.

|  | Medium  control | Amosite Asbestos | Dialead K13D2U Carbon fiber fragments low | CNT1-1 MWCNT low | USRN 20-30 MWCNT low | OCSiAl Tuball SWCNT low | Nanocyl  NC7000 long MWCNT low |
| --- | --- | --- | --- | --- | --- | --- | --- |
| **Group** | **1** | **2** | **3** | **5** | **7** | **9** | **11** |
| **Number of animals examined** | (5) | (5) | (5) | (5) | (5) | (5) | (5) |
| Diaphragm | 0 | 1 | 0 | 0 | 0 | 0 | 0 |
| Spleen | 0 | 2 | 0 | 0 | 0 | 0 | 0 |
| Mesentery | 0 | 0 | 1 | 0 | 0 | 0 | 0 |
