# Supplementary material for: Impact of carbon-based fibers morphologies on their carcinogenic potential
Source: Part Fibre Toxicol. 2026 Feb 7;23:7. doi: 10.1186/s12989-026-00663-y (PMC12931056; doi:10.1186/s12989-026-00663-y)
Supplement: Supplementary file 9 — Supplementary Material 9. [file 12989_2026_663_MOESM9_ESM.docx]

**Supplementary table 8 Neoplasias (non-mesotheliomas) in protocol organs**

|  | Medium  control | Amosite asbestos | Dialed K13D2U Carbonfaser | | CNT1-1 MWCNT | | USRN 20-30 MWCNT | | OCSiAl Tuball SWCNT | | Nanocyl NC7000 MWCNT | |
| --- | --- | --- | --- | --- | --- | --- | --- | --- | --- | --- | --- | --- |
|  |  |  | low | high | low | high | low | high | low | high | low | high |
| **Group** | **1** | **2** | **3** | **4** | **5** | **6** | **7** | **8** | **9** | **10** | **11** | **12** |
| Number of animals examined | 50 | 50 | 50 | 50 | 50 | 50 | 50 | 50 | 50 | 50 | 50 | 50 |
| **Mesentery** |  |  |  |  |  |  |  |  |  |  |  |  |
| Fibroma | 0 | 1 | 0 | 0 | 0 | 0 | 0 | 0 | 0 | 0 | 0 | 0 |
| Hemangioma | 0 | 0 | 0 | 0 | 0 | 0 | 0 | 0 | 0 | 0 | 0 | 1 |
| Liposarcoma | 0 | 0 | 0 | 0 | 0 | 0 | 0 | 0 | 0 | 0 | 1 | 0 |
| Hemangiosarcoma | 0 | 0 | 1 | 0 | 0 | 0 | 0 | 0 | 0 | 0 | 0 | 0 |
| Sarcoma, not otherwise specified - NOS | 0 | 0 | 0 | 0 | 0 | 0 | 0 | 1 | 0 | 0 | 0 | 0 |
| **Spleen** |  |  |  |  |  |  |  |  |  |  |  |  |
| Hemangiosarcoma | 0 | 1 | 1 | 0 | 1 | 0 | 0 | 0 | 0 | 1 | 0 | 0 |
| **Liver** |  |  |  |  |  |  |  |  |  |  |  |  |
| Adenoma, hepatocellular | 0 | 0 | 0 | 3 | 1 | 0 | 3 | 0 | 1 | 0 | 0 | 0 |
| Carcinoma, hepatocellular | 0 | 0 | 2 | 0 | 2 | 2 | 0 | 2 | 0 | 1 | 2 | 1 |
| **Pancreas** |  |  |  |  |  |  |  |  |  |  |  |  |
| Adenoma, islet cell | 4 | 0 | 0 | 3 | 1 | 5 | 4 | 2 | 1 | 2 | 5 | 3 |
| Adenoma, acinar-islet cell | 1 | 0 | 0 | 0 | 0 | 1 | 0 | 0 | 1 | 0 | 0 | 0 |
| Adenoma, acinar cell | 0 | 0 | 0 | 1 | 0 | 1 | 0 | 0 | 0 | 0 | 0 | 0 |
| Adenoma, ductal cell | 0 | 0 | 0 | 0 | 0 | 0 | 0 | 1 | 0 | 0 | 0 | 0 |
| Carcinoma, islet cell | 0 | 1 | 3 | 2 | 0 | 1 | 1 | 1 | 1 | 0 | 3 | 1 |
| Carcinoma, acinar cell | 0 | 0 | 0 | 0 | 0 | 0 | 0 | 0 | 0 | 1 | 0 | 0 |
| **Intestine** |  |  |  |  |  |  |  |  |  |  |  |  |
| Leiomyoma | 0 | 1 | 0 | 0 | 0 | 0 | 0 | 0 | 0 | 0 | 0 | 0 |
| Leiomyosarcoma | 0 | 1 | 0 | 0 | 0 | 0 | 0 | 0 | 0 | 0 | 1 | 0 |
| Malignant schwannoma | 0 | 0 | 0 | 0 | 0 | 1 | 0 | 0 | 0 | 0 | 0 | 0 |
| Adenocarcinoma | 0 | 0 | 0 | 0 | 0 | 0 | 0 | 0 | 1 | 0 | 0 | 0 |
| **Testis** |  |  |  |  |  |  |  |  |  |  |  |  |
| Adenoma, Leydig cell | 2 | 1 | 0 | 1 | 0 | 3 | 0 | 2 | 2 | 3 | 2 | 1 |
| Malignant schwannoma | 0 | 0 | 0 | 0 | 0 | 0 | 0 | 0 | 1 | 0 | 0 | 0 |

MWCNT: multi-walled carbon nanotubes, SWCNT: single-walled carbon nanotubes, low: low dose group; high: high dose group
