# Supplementary material for: Impact of carbon-based fibers morphologies on their carcinogenic potential
Source: Part Fibre Toxicol. 2026 Feb 7;23:7. doi: 10.1186/s12989-026-00663-y (PMC12931056; doi:10.1186/s12989-026-00663-y)
Supplement: Supplementary file 10 — Supplementary Material 10. [file 12989_2026_663_MOESM10_ESM.docx]

**Supplementary Table 9**: Neoplasias (non-mesotheliomas) in non-protocol organs.

|  | Medium  control | Amosite asbestos | Dialead K13D2U Carbon fiber fragments | | CNT1-1 MWCNT | | USRN 20-30 MWCNT | | OCSiAl Tuball SWCNT | | Nanocyl  NC7000 long MWCNT | |
| --- | --- | --- | --- | --- | --- | --- | --- | --- | --- | --- | --- | --- |
|  |  |  | low | high | low | high | low | high | low | high | low | high |
| **Group** | **1** | **2** | **3** | **4** | **5** | **6** | **7** | **8** | **9** | **10** | **11** | **12** |
| Number of animals examined | ∙ | ∙ | ∙ | ∙ | ∙ | ∙ | ∙ | ∙ | ∙ | ∙ | ∙ | ∙ |
| **Non-glandular stomach** |  |  |  |  |  |  |  |  |  |  |  |  |
| Carcinoma, squamous cell | ∙ | 1 | 1 | ∙ | ∙ | ∙ | ∙ | ∙ | ∙ | ∙ | ∙ | ∙ |
| **Kidneys** |  |  |  |  |  |  |  |  |  |  |  |  |
| Hemangioma | ∙ | ∙ | ∙ | ∙ | ∙ | ∙ | ∙ | ∙ | ∙ | ∙ | ∙ | 1 |
| **Abdominal-pelvic adipose tissue** |  |  |  |  |  |  |  |  |  |  |  |  |
| Lipoma | ∙ | ∙ | ∙ | 1 | ∙ | ∙ | ∙ | ∙ | ∙ | ∙ | ∙ | ∙ |
| **Prostate** |  |  |  |  |  |  |  |  |  |  |  |  |
| Adenoma | 1 | ∙ | ∙ | ∙ | ∙ | ∙ | ∙ | ∙ | ∙ | ∙ | ∙ | ∙ |
| Adenocarzinoma | ∙ | ∙ | ∙ | ∙ | ∙ | ∙ | ∙ | ∙ | 1 | ∙ | ∙ | ∙ |
| **Adrenal glands** |  |  |  |  |  |  |  |  |  |  |  |  |
| Adenoma, cortex | ∙ | ∙ | 1 | ∙ | ∙ | ∙ | ∙ | ∙ | ∙ | ∙ | ∙ | 1 |
| Pheochromocytoma, benign | ∙ | ∙ | ∙ | 1 | ∙ | ∙ | ∙ | ∙ | ∙ | ∙ | ∙ | ∙ |
| Pheochromocytoma, complex, benign | ∙ | ∙ | 1 | ∙ | ∙ | ∙ | ∙ | ∙ | ∙ | ∙ | ∙ |  |
| Carcinoma, cortex | ∙ | 1 | ∙ | ∙ | ∙ | ∙ | ∙ | ∙ | 1 | ∙ | ∙ | ∙ |
| Pheochromocytoma, malignant | ∙ | ∙ | ∙ | ∙ | ∙ | ∙ | ∙ | 1 | ∙ | ∙ | ∙ | ∙ |
| Pheochromocytoma, complex, malignant | 1 | ∙ | 1 | ∙ | ∙ | ∙ | ∙ | ∙ | ∙ | ∙ | ∙ | 1 |
| **Thyroid glands** |  |  |  |  |  |  |  |  |  |  |  |  |
| Adenoma, C-cell | ∙ | ∙ | ∙ | ∙ | 1 | ∙ | ∙ | ∙ | ∙ | ∙ | ∙ | ∙ |
| Adenoma, follicular cell | ∙ | 3 | ∙ | 2 | 2 | 1 | 2 | 1 | 1 | ∙ | ∙ | ∙ |
| Carcinoma, C-cell | ∙ | ∙ | ∙ | 1 | ∙ | ∙ | ∙ | ∙ | ∙ | ∙ | ∙ | ∙ |
| Carcinoma, follicular cell | 2 | ∙ | ∙ | ∙ | ∙ | 1 | ∙ | ∙ | ∙ | 2 | 2 | ∙ |
| **Parathyroid glands** |  |  |  |  |  |  |  |  |  |  |  |  |
| Adenoma | ∙ | ∙ | 1 | ∙ | 1 | ∙ | ∙ | ∙ | ∙ | ∙ | ∙ | ∙ |
| **Pituitary gland** |  |  |  |  |  |  |  |  |  |  |  |  |
| Adenoma, pars distalis | 2 | 6 | 3 | 3 | 6 | 5 | 8 | 5 | 3 | 3 | 6 | 3 |
| Adenoma, pars intermedia | ∙ | 1 | ∙ | ∙ | ∙ | ∙ | ∙ | ∙ | ∙ | ∙ | ∙ | ∙ |
| Carcinoma, pars distalis | ∙ | 3 | 1 | ∙ | ∙ | 1 | ∙ | ∙ | ∙ | 2 | ∙ | 1 |
| **Hematolymphoid system** |  |  |  |  |  |  |  |  |  |  |  |  |
| Lymphoma | 1 | ∙ | 3 | ∙ | 3 | 6 | 1 | 1 | 1 | 0 | 2 | 1 |
| Leukemia, not otherwise specified | ∙ | 1 | ∙ | ∙ | ∙ | ∙ | ∙ | ∙ | ∙ | 1 | ∙ | 1 |
| Histiocytic Sarcoma | ∙ | ∙ | ∙ | ∙ | 1 | ∙ | ∙ | 1 | ∙ | 1 | ∙ | 1 |
| **Mesenteric lymph nodes** |  |  |  |  |  |  |  |  |  |  |  |  |
| Hemangioma | 2 | ∙ | 2 | ∙ | ∙ | 1 | ∙ | 1 | 1 | 1 | ∙ | 1 |
| Hemangiosarcoma | 1 | ∙ | 1 | 1 | 3 | 1 | ∙ | 3 | 2 | 2 | 1 | 3 |
| Angiosarcoma | ∙ | ∙ | ∙ | 1 | ∙ | ∙ | ∙ | ∙ | 1 | ∙ | ∙ | ∙ |
| **Lung-associated lymph nodes** |  |  |  |  |  |  |  |  |  |  |  |  |
| Hemangioma | ∙ | 1 | ∙ | ∙ | ∙ | ∙ | ∙ | ∙ | ∙ | ∙ | ∙ | ∙ |
| Hemangiosarcoma | ∙ | ∙ | ∙ | ∙ | ∙ | ∙ | ∙ | ∙ | ∙ | ∙ | ∙ | 1 |
| **Lymph nodes, not otherwise specified** |  |  |  |  |  |  |  |  |  |  |  |  |
| Hemangioma | ∙ | ∙ | ∙ | ∙ | ∙ | ∙ | ∙ | ∙ | ∙ | ∙ | ∙ | 1 |
| Hemangiosarcoma | 1 | 1 | ∙ | ∙ | ∙ | ∙ | ∙ | ∙ | ∙ | ∙ | ∙ | ∙ |
| **Thymus** |  |  |  |  |  |  |  |  |  |  |  |  |
| Thymoma, benign | 1 | ∙ | ∙ | 3 | ∙ | 1 | ∙ | 1 | ∙ | 1 | ∙ | 1 |
| **Brain** |  |  |  |  |  |  |  |  |  |  |  |  |
| Astrocytoma, malignant | ∙ | 1 | ∙ | ∙ | ∙ | ∙ | ∙ | ∙ | 1 | ∙ | ∙ | ∙ |
| Granular cell tumor, malignant | ∙ | ∙ | ∙ | ∙ | ∙ | ∙ | ∙ | 1 | ∙ | ∙ | 1 | ∙ |
| Meningioma, malignant | ∙ | ∙ | ∙ | ∙ | ∙ | ∙ | ∙ | ∙ | ∙ | ∙ | ∙ | 1 |
| **Epiphysis** |  |  |  |  |  |  |  |  |  |  |  |  |
| Pinealoma, benign | ∙ | ∙ | 1 | ∙ | ∙ | ∙ | ∙ | 1 | ∙ | ∙ | ∙ | ∙ |
| **Oral cavity** |  |  |  |  |  |  |  |  |  |  |  |  |
| Carcinoma, squamous cell | 1 | ∙ | ∙ | ∙ | ∙ | ∙ | ∙ | ∙ | ∙ | ∙ | ∙ | ∙ |
| **Salivary glands** |  |  |  |  |  |  |  |  |  |  |  |  |
| Adenocarcinoma | ∙ | ∙ | 1 | ∙ | ∙ | ∙ | ∙ | 1 | ∙ | 1 | ∙ | ∙ |
| Tumor, mixed, malignant | ∙ | ∙ | ∙ | ∙ | ∙ | ∙ | ∙ | ∙ | ∙ | ∙ | 1 | ∙ |
| **Lunge** |  |  |  |  |  |  |  |  |  |  |  |  |
| Carcinoma, bronchiolo-alveolar | ∙ | ∙ | ∙ | ∙ | ∙ | 1 | ∙ | ∙ | 1 | ∙ | ∙ | ∙ |
| **Skin** |  |  |  |  |  |  |  |  |  |  |  |  |
| Tumor, hair follicle, benign | 1 | 1 | 1 | 2 | 4 | 4 | 2 | 3 | 4 | 5 | 4 | 4 |
| Tumor, basal cell, benign | ∙ | ∙ | ∙ | ∙ | 1 | ∙ | ∙ | ∙ | ∙ | ∙ | ∙ | ∙ |
| Keratoacanthoma | 2 | 1 | ∙ | ∙ | ∙ | ∙ | ∙ | 1 | 1 | 1 | 1 | ∙ |
| Papilloma | ∙ | 1 | ∙ | 1 | ∙ | ∙ | ∙ | ∙ | ∙ | ∙ | ∙ | 2 |
| Adenoma, sebaceous cell | ∙ | ∙ | ∙ | ∙ | ∙ | ∙ | 2 | ∙ | ∙ | ∙ | 1 | ∙ |
| Carcinoma, squamous cell | ∙ | 1 | 2 | ∙ | ∙ | ∙ | 2 | ∙ | ∙ | 1 | ∙ | 1 |
| Carcinoma, sebaceous cell | 1 | ∙ | ∙ | 2 | ∙ | ∙ | ∙ | ∙ | ∙ | ∙ | ∙ | ∙ |
| **Subcutis** |  |  |  |  |  |  |  |  |  |  |  |  |
| Fibroma | ∙ | ∙ | 2 | ∙ | 2 | 1 | 1 | 1 | 1 | 2 | 1 | 2 |
| Granular cell tumor, benign | 1 | 1 | ∙ | ∙ | ∙ | ∙ | ∙ | ∙ | ∙ | ∙ | ∙ | ∙ |
| Lipoma | ∙ | ∙ | ∙ | ∙ | ∙ | 1 | ∙ | ∙ | 1 | ∙ | 1 | ∙ |
| Fibrosarcoma | ∙ | ∙ | ∙ | ∙ | 1 | ∙ | ∙ | ∙ | 2 | 2 | ∙ | ∙ |
| Fibrosarcoma, pleomorphic | ∙ | ∙ | ∙ | ∙ | ∙ | ∙ | ∙ | 1 | ∙ | ∙ | ∙ | ∙ |
| Hemangiosarcoma | ∙ | 2 | 2 | 2 | ∙ | ∙ | ∙ | ∙ | 1 | ∙ | ∙ | ∙ |
| Liposarcoma | ∙ | ∙ | ∙ | 1 | ∙ | ∙ | ∙ | ∙ | ∙ | ∙ | ∙ | ∙ |
| Schwannoma, malignant | ∙ | 1 | ∙ | 2 | 1 | ∙ | ∙ | ∙ | 1 | ∙ | ∙ | ∙ |
| Sarcoma, not otherwise specified - NOS | ∙ | ∙ | ∙ | ∙ | 1 | ∙ | ∙ | ∙ | ∙ | 1 | ∙ | ∙ |
| **Mammary gland** |  |  |  |  |  |  |  |  |  |  |  |  |
| Fibroadenoma | ∙ | ∙ | ∙ | ∙ | 1 | ∙ | 1 | ∙ | ∙ | ∙ | ∙ | ∙ |
| Fibroma | 1 | ∙ | ∙ | ∙ | ∙ | ∙ | ∙ | ∙ | ∙ | ∙ | ∙ | ∙ |
| Adenocarcinoma | ∙ | ∙ | ∙ | 1 | ∙ | ∙ | ∙ | ∙ | ∙ | ∙ | ∙ | ∙ |
| **Zymbal glands** |  |  |  |  |  |  |  |  |  |  |  |  |
| Carcinoma, sebaceous cell | ∙ | ∙ | ∙ | ∙ | ∙ | ∙ | ∙ | ∙ | 1 | ∙ | ∙ | ∙ |
| **Preputial glands** |  |  |  |  |  |  |  |  |  |  |  |  |
| Carcinoma, squamous cell | ∙ | ∙ | ∙ | ∙ | ∙ | ∙ | ∙ | ∙ | ∙ | 1 | ∙ | ∙ |
| **Skeletal muscle** |  |  |  |  |  |  |  |  |  |  |  |  |
| Fibrosarcoma | ∙ | ∙ | 1 | ∙ | 2 | ∙ | ∙ | ∙ | 1 | ∙ | ∙ | 1 |
| Hemangiosarcoma | ∙ | ∙ | ∙ | ∙ | ∙ | 1 | 1 | ∙ | 1 | ∙ | ∙ | 2 |
| **Bone** |  |  |  |  |  |  |  |  |  |  |  |  |
| Osteosarcoma | 2 | ∙ | 1 | ∙ | 1 | ∙ | ∙ | ∙ | 1 | ∙ | ∙ | ∙ |

MWCNT: multi-walled carbon nanotubes, SWCNT: single-walled carbon nanotubes, low: low dose group; high: high dose group
