# Supplementary material for: Impact of carbon-based fibers morphologies on their carcinogenic potential
Source: Part Fibre Toxicol. 2026 Feb 7;23:7. doi: 10.1186/s12989-026-00663-y (PMC12931056; doi:10.1186/s12989-026-00663-y)
Supplement: Supplementary file 11 — Supplementary Material 11. [file 12989_2026_663_MOESM11_ESM.docx]

**Supplementary table 10**: Diaphragmatic thickness measurement (µm).

|  | Medium  control | Amosite asbestos | Dialead K13D2U fragments low | CNT1-1 MWCNT low | USRN 20-30 MWCNT low | OCSiAl Tuball SWCNT low | Nanocyl NC7000 long MWCNT low |
| --- | --- | --- | --- | --- | --- | --- | --- |
| **Group** | **1** | **2** | **3** | **5** | **7** | **9** | **11** |
| Diaphragm thickness (µm) | **7.0** | **9.2** | **5.5** | **6.8** | **8.9** | **5.8** | **6.0** |
| Standard deviation | **2.3** | **4.8** | **1.5** | **1.6** | **2.5** | **1.2** | **1.8** |

MWCNT: multi-walled carbon nanotubes, SWCNT: single-walled carbon nanotubes, low: low dose group
